# Proteomic profiling reveals growth condition-driven functional differences in bacterial membrane vesicles from planktonic and biofilm cultures

**DOI:** 10.3389/fcimb.2026.1881620

**Published:** 2026-10-01

**Authors:** Nathalie Jung, Lennart Christe, Stefanie Gier, Jana Romy Friedrich, Fadi Haddad, Pauline Roth, João Mello-Viera, Thorsten Mosler, Stefan Schiller, Achilleas S. Frangakis, Ivan Đikić, Maike Windbergs

**Affiliations:** 1 Institute of Pharmaceutical Technology, Goethe University Frankfurt, Frankfurt am Main, Germany; 2 Buchmann Institute for Molecular Life Sciences and Institute for Biophysics, Goethe University Frankfurt, Frankfurt am Main, Germany; 3 Molecular Signaling Group, Buchmann Institute for Molecular Life Sciences, Goethe University Frankfurt, Frankfurt am Main, Germany; 4 Molecular Signaling Group, Institute of Biochemistry II, Medical Faculty, Goethe University Frankfurt, Frankfurt am Main, Germany

**Keywords:** bacterial membrane vesicles, biofilm, BMV, host-pathogen interaction, planktonic growth, proteomics, *Pseudomonas aeruginosa*, *Staphylococcus aureus*

## Abstract

**Introduction:**

Bacterial membrane vesicles (BMVs) are key mediators of host-pathogen interaction and intercellular communication. However, their characterization has been conducted almost exclusively under planktonic growth conditions, even though biofilm formation is the predominant mode of bacterial persistence in chronic and device-associated infections. How the shift from planktonic to biofilm growth reshapes BMV composition and function, and how this is affecting the host, remains poorly understood.

**Methods:**

Here, we systematically compared BMVs from the ESKAPE pathogens *Staphylococcus aureus* and *Pseudomonas aeruginosa*, representing Gram-positive and Gram-negative bacteria, across both growth conditions. Particle sizes and number-weighted size distributions were obtained by dynamic light scattering and nanoparticle tracking analysis, respectively.

**Results:**

Cryogenic electron micrographs showed intact, membrane-enclosed vesicles for all four isolations. Biofilm-derived BMVs exhibited a more negative surface charge compared to their planktonic counterparts. Quantitative proteomics identified growth mode as a strong determinant of cargo: planktonic BMVs were enriched in ribosomal proteins, whereas biofilm-derived BMVs carried central metabolic enzymes. Notably, exopolysaccharide biosynthesis proteins of the Psl, Pel, and Ica systems were more abundant in planktonic than in biofilm BMVs. *P. aeruginosa* BMVs isolated from biofilms carried heme and iron acquisition systems, proteins of the oxidative stress defense, and the secreted proteases LasB and AprA, while planktonic *S. aureus* BMVs were enriched in secreted virulence factors, highlighting species-level differences. Functionally, all preparations reduced metabolic activity in epithelial cells with only limited lactate dehydrogenase release, identifying metabolic suppression rather than membrane rupture as the dominant effect. In the case of *P. aeruginosa*, biofilm-derived BMVs suppressed metabolic activity significantly more than planktonic ones. In human macrophages, planktonic BMVs elicited the strongest IL-1β and TNF-α responses, whereas biofilm-derived vesicles induced earlier secretion of IL-6 and, for *P. aeruginosa*, a distinctly reduced release of the anti-inflammatory cytokine IL-10. These effects persisted under polymyxin B, which reduced but did not abolish the overall cytokine responses.

**Discussion:**

This indicates that vesicle cargo does not simply mirror the biofilm matrix, pointing to a contribution of vesicle-associated components beyond lipopolysaccharide. Together, these data establish a comparative framework linking bacterial growth mode to BMV composition and host cell response, advancing the understanding of vesicle-mediated processes in chronic infection.

## 1 Introduction

Bacterial infections remain a major global health burden, particularly in their chronic manifestations, where conventional antimicrobial therapies frequently fail to achieve full eradication (Lu et al., 2014; Ahmed and Baptiste, 2018; Ali Alghamdi et al., 2023). The ESKAPE pathogens, *Enterococcus faecium*, *Staphylococcus aureus*, *Klebsiella pneumoniae*, *Acinetobacter baumannii*, *Pseudomonas aeruginosa*, and *Enterobacter* species, are among the most clinically problematic, collectively responsible for a large proportion of antibiotic-resistant nosocomial infections worldwide (Ali Alghamdi et al., 2023). A key factor in their persistence is their capacity to form biofilms, structured multicellular communities enclosed within a self-produced matrix, which provides a protective niche that shields bacteria from antibiotic penetration and host immune clearance (Khelissa et al., 2019; Mirghani et al., 2022). Consequently, biofilm-associated infections are notoriously difficult to treat and are a hallmark of many device-associated infections, chronic wounds, and respiratory and bloodstream infections (Chakraborty et al., 2021; Pinto et al., 2021; Mirghani et al., 2022; Cavallo et al., 2024).

Biofilm and planktonic growth modes represent physiologically distinct states, each associated with different metabolic, regulatory, and virulence strategies (Spoering and Lewis, 2001; Hernández-Jiménez et al., 2013). Within the complex architecture of biofilms, intercellular communication and coordination are essential for survival and pathogenicity. Beyond well-established mechanisms such as quorum sensing, bacterial membrane vesicles (BMVs) have emerged as critical mediators of bacterial interactions within communities and in host-pathogen interactions. The release of such vesicles is conserved across Gram-negative and Gram-positive bacteria under various growth conditions, including planktonic cultures and biofilm-associated colonies (Tashiro et al., 2017; Toyofuku et al., 2023; Jeong et al., 2024). BMVs can carry diverse cargo, including proteins, lipids, and nucleic acids, and serve as vehicles for delivering virulence factors (Cecil et al., 2019; Juodeikis and Carding, 2022; Toyofuku et al., 2023). They can effectively overcome biological barriers within the human body, modulate host immune responses, and reshape the local microenvironment (Bauman and Kuehn, 2006, 2009; Im et al., 2017). Despite extensive studies in planktonic cultures, the role of BMVs in biofilm biology and their contribution to chronic infections remain poorly understood (Hernández-Jiménez et al., 2013; De Langhe et al., 2024). This gap is particularly consequential given that biofilm-resident bacteria, including clinically relevant species such as *P. aeruginosa* and *S. aureus*, exist in a fundamentally different physiological state than their planktonic counterparts. Thus, BMVs produced by biofilm-resident bacteria may differ from those released during planktonic growth in terms of composition, biophysical properties, and functional activity. Such differences could render biofilm-derived BMVs a distinct and underappreciated mechanism by which bacteria interact with the host independently of direct cellular contact, potentially contributing to the establishment and persistence of chronic infections (Spencer and Yeruva, 2020; Battista et al., 2025). A deeper understanding of how bacterial growth shapes BMV biology may therefore reveal new insights into infection pathogenesis and into the biology of vesicle-mediated communication.

In this study, we present a comparative analysis of BMVs isolated from planktonic and biofilm cultures of *S. aureus* and *P. aeruginosa*, representative Gram-positive and Gram-negative ESKAPE pathogens, respectively. Vesicle composition was analyzed using quantitative proteomics to deconvolute the vesicular proteome, which was further complemented by monitoring the cellular effects of BMVs in human epithelial cell lines and macrophage monocultures, thereby providing valuable insights into their functional behavior. Together, these analyses provide a comparative, descriptive framework for how bacterial growth conditions shape BMV composition and host cell responses in chronic infection.

## 2 Materials and methods

### 2.1 Cultivation of planktonic cultures

Single colonies of *Pseudomonas aeruginosa* (ATCC 27853) and *Staphylococcus aureus* (ATCC 29213) were suspended in 2 mL phosphate-buffered saline (PBS, Biowest, Nuaillé, France) to achieve turbidity equivalent to the 0.5 McFarland standard. Subsequently, a pre-culture was prepared in nutrient broth medium (NB, Becton Dickinson GmbH, Heidelberg, Germany) using a standardized inoculum and incubated at 37°C (180 rpm). After 24 h, main cultures of 500 mL starting at an optical density (OD_600_) of 0.05 were grown until the early stationary phase of bacterial growth was reached (12 h for *S. aureus* and 17 h for *P. aeruginosa*), at which point BMVs were isolated.

### 2.2 Electrospinning of scaffolds and biofilm cultivation

Fiber scaffolds for biofilm cultivation were prepared as previously described (Wächter et al., 2023). Briefly, 20wt% of cellulose acetate (Mn 30,000, Merck KGaA, Darmstadt, Germany) or gelatin (from porcine skin, 300 Bloom, type A, Sigma-Aldrich, Steinheim, Germany) were dissolved in 80% glacial acetic acid and stirred overnight. Equal volumes of the polymer solutions were then combined and homogenized using a dual centrifuge (Zentrimix 380 R, Andreas Hettich GmbH & Co. KG, Tuttlingen, Germany) with 10 zirconia beads per tube at 1,800 rpm for 2 min at 4°C. The solution was sonicated and loaded into a syringe (B. Braun, Melsungen, Germany) equipped with a 27-gauge blunt-tip needle. Electrospinning was performed using a voltage of 16-18 kV and a flow rate of 1.4 mL/h at 27°C and 20% relative humidity. The resulting fibers were collected on a rotating drum (10 cm diameter, 150 rpm) and stored in a desiccator at room temperature (RT) until further use. Biofilm cultivation was performed according to an established protocol (Wächter et al., 2023). Fiber punches (12 mm diameter) were prepared, placed onto nutrient agar plates (Thermo Fisher Scientific, Dreieich, Germany) supplemented with 20% fetal calf serum (FCS, Sigma-Aldrich, Steinheim, Germany), and inoculated with a *P. aeruginosa* or *S. aureus* suspension, respectively. Biofilms were cultured at 37°C for 48 or 72 h for *P. aeruginosa* and *S. aureus*, respectively, to reach full maturation. Biofilms were transferred onto new agar plates every 24 h.

### 2.3 Scanning electron microscopy

For structural analysis, biofilm samples were fixed in 4% formaldehyde for 6 h, then dehydrated in a graded ethanol series (50%, 70%, 96%, and 100% ethanol). After critical-point drying to preserve morphology, samples were sputter-coated (SC7620, Quantum Design GmbH, Darmstadt, Germany) with gold/palladium for 3 min to prevent charging during imaging. SEM (Evo 10, Carl Zeiss Microscopy GmbH, Jena, Germany) analysis was performed at 8 kV, with samples mounted on carbon tape.

### 2.4 Histology

To confirm uniform colonization of fiber scaffolds by bacteria, biofilm models were fixed in 4% formaldehyde for 6 h, washed with PBS, and stored at 4°C. The samples were dehydrated in an ethanol series (70%, 80%, 96%) and embedded in glycol methacrylate resin (Technovit 7100, Kulzer GmbH, Hanau, Germany). Resin blocks were sectioned longitudinally through the biofilm at 5 µm thickness using a microtome (Cut 6062, SLEE medical GmbH, Nieder-Olm, Germany) and mounted on glass slides. Hematoxylin (Gill III, Morphisto GmbH, Offenbach, Germany) staining was performed by immersing the slides in the staining solution for 30 min, followed by thorough washing and drying. Cover slips were embedded, and imaging was performed via bright-field microscopy (LSM900, Carl Zeiss Microscopy, Jena, Germany).

### 2.5 Isolation of bacterial membrane vesicles

BMVs of planktonic cultures were harvested at early stationary phase, as confirmed by optical density measurements. Per isolation, 500 mL of culture were processed. Bacteria were pelleted (15,000 × g for 10 min at 4°C), the supernatant was sterile-filtered (0.22 µm) and concentrated to approximately 10 mL using tangential flow filtration (Vivaflow 200, 100 kDa MWCO, Sartorius AG, Göttingen, Germany), followed by ultracentrifugation (Optima XPN-80, Beckman Coulter, Krefeld, Germany) at 150,000 × g for 2 h at 4° C. The vesicle pellet was washed and resuspended in 250 µL PBS. Samples were stored at 4°C, and further experiments were conducted within 24 h. For BMV isolation from biofilm-associated bacteria, biofilms were transferred into 1.8 mL tubes containing 1mL PBS and 10 zirconia beads (four scaffolds per tube). Disruption of the biofilm matrix was achieved using a bead beater set to 1.4 m/s, 2 × 1 min with a 15 s interval (Bead Mill Max, VWR International, Darmstadt, Germany). The resulting suspensions were combined, and biomass, along with beads and fiber remnants, was pelleted by centrifugation at 15,000 × g for 10 min at 4°C. The supernatant was sterile-filtered (0.22 µm), and BMVs were isolated via ultracentrifugation (Optima XPN-80, Beckman Coulter, Krefeld, Germany) at 150,000 × g for 2 h at 4°C. After two washing steps with sterile, vesicle-free PBS, the vesicle-containing pellet was resuspended in 250 µL PBS and stored at 4°C for a maximum of 24 h. Protein concentrations were determined via a bicinchoninic acid (BCA) assay according to the manufacturer’s instructions (Thermo Fisher Scientific, Dreieich, Germany).

### 2.6 Determination of colony-forming units

Potential bacterial lysis during biofilm disruption was evaluated in a split-sample design. Three independent overnight cultures per bacterial strain were adjusted to an OD_600_ of 0.5 and subsequently divided into two aliquots (N = 3). One aliquot was supplemented with 10 zirconia beads and subjected to bead-beating (1.4 m/s, 2 × 1 min, 15 s interval, Bead Mill Max, VWR International, Darmstadt, Germany), and one was left as an untreated parallel control. To evaluate CFU of biofilm-associated bacteria, four biofilm scaffolds were pooled per replicate and resuspended in 1 mL PBS (N = 3). CFU was determined using an adapted drop-plate method (Miles et al., 1938). Ten-fold serial dilutions were prepared in 96-well plates, yielding dilutions of 10^-1^ to 10^-12^. Three technical drops of 10µL each were plated onto NB agar plates per dilution and biological replicate (n = 3). After 16 h incubation at 37°C, bacterial colonies were counted, and CFU/mL was calculated as ΣN/(k × V × d), with ΣN as the summed colony count across technical drops, k = 3 drops, V = 0.01 mL, and d the dilution factor. Counts were analyzed on a log_10_ scale to account for approximately log-normally distributed bacterial densities. Values are displayed as back-transformed geometric means of log_10_ values, with standard deviations calculated on the log_10_ scale.

### 2.7 Hydrodynamic diameter and zeta potential measurement

The hydrodynamic diameter of BMVs was determined by dynamic light scattering (DLS) (Zetasizer NanoZS, Malvern Panalytical, Malvern, UK). Measurements were conducted in 1mL polystyrene cuvettes, and the hydrodynamic diameter was determined based on intensity distributions. Samples were measured immediately after isolation and after overnight storage at 4°C. Hydrodynamic diameters and surface charge were determined for four independent isolations per sample group (N = 4) and measured in triplicate (n = 3). Zeta potential characterization of BMVs was performed by measuring their electrophoretic mobility using electrophoretic light scattering (ELS) (Zetasizer NanoZS, Malvern Panalytical, Malvern, UK) in a dip cell configuration. Samples were measured immediately after isolation and after overnight storage at 4°C.

### 2.8 Cryogenic transmission electron microscopy

For each BMV sample, three independent biological replicates were combined into a single preparation (equal volume), and further concentrated using Amicon™ Ultra centrifugal filters (100 kDa MWCO, Millipore, Merck KGaA, Darmstadt, Germany) to ensure adequate particle concentration per grid. A 3.5 µL droplet was applied to a 40 s glow-discharged Quantifoil R1.2/1.3 Cu 300 mesh grid (Electron Microscopy Sciences, Hatfield, USA) coated with a 3.0 nm continuous carbon layer. The grids were plunge-frozen in liquid ethane using a Vitrobot Mark IV (Thermo Fisher Scientific, Dreieich, Germany) at 100% relative humidity (20°C), with a nominal blot force of -3, a wait time of 5 s, and a drain time of 0.5 s; blotting time was set at 10-20 s. Whatman 595 filter papers for blotting were equilibrated in the Vitrobot chamber for 1 h at 100% relative humidity and 4°C before use. Dose-fractionated movies were collected using SerialEM v4.2.21 at a nominal magnification of 53,000 × (1.717 Å per pixel) in nanoprobe mode on a Titan Krios G3i transmission electron microscope (Thermo Fisher Scientific, Dreieich, Germany) operated at 300 kV and equipped with a GIF Quantum S.E. post-column energy filter in zero-loss peak mode and a K3 direct electron detector (Gatan, Inc., Pleasanton, USA) (Mastronarde, 2003). Each micrograph was recorded as a 50-frame movie with a total dose of 50 e^-^/Å^2^. The camera was operated in counting mode at a dose rate of ~19.1 e^-^/px/s. Data were collected with a defocus of -3 µm. Beam-induced motion correction and CTF estimation were performed in CryoSPARC (Punjani et al., 2017). Images were 4 × binned during processing.

### 2.9 Nanoparticle tracking analysis

Particle size distribution and particle concentration of the isolated BMVs were determined by nanoparticle tracking analysis using a NanoSight Pro device (Malvern Panalytical, Malvern, United Kingdom) equipped with a 488 nm laser. NTA was performed using an aliquot of the same combined BMV preparations used for cryo-TEM before further concentration (see 2.8). Each sample was diluted to an appropriate concentration in sterile, particle-free PBS within the linear range of the instrument (final dilution factor: 2 and 4 for *P. aeruginosa* and *S. aureus* BMVs, respectively) and loaded into the measuring cell using a syringe pump. For each measurement, videos were captured with a static setup (flow rate of 0.0 µL/min), using the following specifications: T = 29.4°C, capture duration 11.5 s, and 750 frames; samples were pushed through manually after each measurement. Data were further analyzed via NS Xplorer software (v2.0). Each preparation was measured in five captures, and the reported errors reflect the standard deviation across these captures (n = 5).

### 2.10 Protein extraction and proteome analysis

Proteome analysis of isolated BMVs was conducted via mass spectrometry (MS). For this purpose, BMVs were lysed in low-binding tubes with lysis buffer (2% SDS, 50 mM Tris pH 8.5, 10 mM TCEP, 40 mM CAA), supplemented with protease inhibitor cocktail and phosphatase inhibitors, followed by heating at 95°C for 10 min, sonication for 2 min, and a second incubation at 95°C for 5 min. Proteins were precipitated using methanol/chloroform. The protein-containing interphase was washed with methanol, vortexed, and centrifuged at maximum speed for 10 min at 4°C. The protein pellet was dried, resuspended in 100 µL digestion buffer (8 M urea, 50 mM Tris pH 8.5), and incubated for 10 min at RT at 750 rpm. Protein content was quantified via BCA assay according to the manufacturer’s instructions (Thermo Fisher Scientific, Dreieich, Germany). Enzymatic digestion was performed with Lys-C (Wako Chemicals, Neuss, Germany) and trypsin (Promega, Walldorf, Germany) (1 µg/50 µg and 1 µg/100 µg of protein, 1:50 w/w and 1:100 w/w, respectively) overnight at 37°C under constant shaking (550 rpm). Digestion was stopped with 1% trifluoroacetic acid, and samples were purified by solid-phase extraction using C18 SepPak columns (Waters Corporation, Eschborn, Germany). Peptide concentrations were quantified using microBCA (Thermo Fisher Scientific, Dreieich, Germany).

### 2.11 Liquid chromatography-mass spectrometry analysis

Dried peptides were resuspended in 2% acetonitrile with 0.1% trifluoroacetic acid prior to LC-MS/MS analysis on a QExactive HF mass spectrometer coupled to an easy nLC 1200 (Thermo Fisher Scientific, Dreieich, Germany) fitted with a 35 cm long, 75 μm ID fused-silica column packed in-house with 1.9 μm C18 particles (Reprosil pur, Dr. Maisch HPLC GmbH, Ammerbruch-Entringen, Germany). The column was maintained at 50°C using an integrated column oven (Sonation GmbH, Biberach, Germany). HPLC solvents consisted of 0.1% formic acid in water (Buffer A) and 0.1% formic acid/80% acetonitrile in water (Buffer B). Peptides were eluted in a non-linear gradient of 4-35% Buffer B over 60 min, followed by a stepwise increase to 95% Buffer B in 6 min, which was held for another 9 min and directly sprayed into the mass spectrometer equipped with a nanoFlex ion source (Thermo Fisher Scientific, Dreieich, Germany). Full-scan MS spectra (300-1,650*m/z*) were acquired in profile mode at a resolution of 60,000 at *m/z* 200, a maximum injection time of 25 ms, and an automatic gain control (AGC) target of 3 × 10^6^. Up to 15 of the most intense peptides per full scan were isolated using a 1.4-Th window for fragmentation by higher-energy collisional dissociation (normalized collision energy of 27). MS/MS spectra were acquired in profile mode with a resolution of 15,000, a maximum injection time of 25 ms, and an AGC target value of 1 × 10^5^. Only ions with a charge state between 2 and 5 were considered for fragmentation.

### 2.12 LC-MS data processing

MS raw data processing was performed with MaxQuant (v1.6.17.0) and its built-in label-free quantification algorithm MaxLFQ, applying default parameters (Tyanova et al., 2016). Acquired spectra were searched against the *P. aeruginosa* proteome (Taxonomy ID 208964) downloaded from UniProt (09-04-2023; 5564 sequences) or the *S. aureus* proteome (Taxonomy ID 93061) downloaded from UniProt (09-04-2023; 2889 sequences) and a collection of common contaminants using the Andromeda search engine integrated in MaxQuant (Cox et al., 2011). Identifications were filtered to obtain false discovery rates (FDR) below 1% for both peptide spectrum matches (PSM; minimum length of 7 amino acids) and proteins using a target-decoy strategy (Elias and Gygi, 2007). After filtering out protein groups with fewer than 2 of 3 valid values within a group, missing values were imputed using the “Replace missing values from normal distribution” function in Perseus (v2.0.11) and replaced with randomly sampled values from a downshifted, narrowed normal distribution to represent low-abundance proteins below the limit of detection. Log_2_ intensities were normalized by z-scoring before p-values were derived by Student’s t-test and adjusted for multiple comparisons by Benjamini-Hochberg correction. Proteins with a difference in z-scored log_2_ intensity >1 or <-1 and an adjusted p-value < 0.05 were deemed enriched. The mass spectrometry proteomics data have been deposited in the ProteomeXchange Consortium via the PRIDE partner repository with the dataset identifier PXD078302 (Perez-Riverol et al., 2025). Principal component analysis (PCA) was performed to assess overall sample variation and identify patterns between the experimental conditions. Using log_2_-transformed, z-score-standardized intensity data, PCA was performed in R with scaling disabled. The variance explained by each principal component was calculated as the squared standard deviation of that component, expressed as a percentage of the total variance. The first six principal components were used to calculate the percentage of variance explained. For visualization, the first two principal components (PC1 and PC2), which explain the majority of the total variance, were plotted in a scatter plot.

### 2.13 Evaluation of cytotoxic potential

Cytotoxicity of BMVs was assessed in the two human epithelial cell lines Caco-2 (clone C2BBe1, passages 50-62, LGC Standards, Teddington, UK) and HaCaT (passages 45-47, CLS Cell Lines Service GmbH, Eppelheim, Germany). Cells were seeded at a density of 30,000 cells per well in a 96-well plate in 100 µL of Dulbecco’s Modified Eagle Medium (DMEM, Gibco, Carlsbad, US) supplemented with 10% FCS; for Caco-2 cells, the medium was further supplemented with 1% non-essential amino acids (NEAA, Gibco, Carlsbad, US). Cells were cultivated for 24 h prior to treatment. For both cell lines, BMVs were diluted in PBS to final concentrations of 10, 25, 50, and 100 µg/mL protein in cell culture medium; buffer-treated cells served as negative controls. After treatment, cells were incubated for 24 h at 37°C with 5% CO_2_. Metabolic activity was determined by 3-(4,5-dimethylthiazol-2-yl)-2,5-diphenyltetrazolium bromide (MTT, Sigma-Aldrich, Steinheim, Germany) assay. Briefly, the supernatant was discarded, MTT reagent (1 mg/mL in cell culture medium) was added, and cells were incubated for an additional 4 h. The resulting formazan crystals were dissolved in 100 µL dimethyl sulfoxide per well, and absorbance was measured at 570 nm using a microplate reader (Tecan Spark, Männedorf, Switzerland). Metabolic activity was calculated using the following formula and is expressed as a percentage of the negative control:


$$Cell\;viability\;[\%]=\frac{\left(A_{sample}-Blank\right)}{\left(A_{negative\;Ctrl}-Blank\right)}\times 100$$

MTT assays were performed with three independent BMV isolations per sample group (N = 3), each measured in three technical replicates (n = 3).

### 2.14 Lactate dehydrogenase release assay

To complement the metabolic activity readout with plasma membrane integrity, lactate dehydrogenase (LDH) release into the supernatant was quantified via a cytotoxicity detection kit (11644793001, Roche, Merck KGaA, Darmstadt, Germany) according to the manufacturer’s instructions. Caco-2 and HaCaT cells were seeded and treated with BMVs (10, 25, 50, and 100 µg/mL total protein) as described for the MTT assay. PBS-treated cells served as a negative control, whereas cells treated with Triton X-100 (1% in cell culture medium) served as a positive control. After 24 h, 90 µL of cell-free supernatant were transferred to a fresh plate and incubated with an equal volume of the reaction mixture for 20 min at RT in the dark. Absorbance was measured at 492 nm with reference at 620 nm using a microplate reader (Tecan Spark, Männedorf, Switzerland). LDH release was calculated with the following formula:


$$LDH\;release\;[\%]=\frac{\left(A_{sample}-A_{negative\;Ctrl}\right)}{\left(A_{positive\;Ctrl}-A_{negative\;Ctrl}\right)}\times 100$$

Values below the spontaneous LDH release of the negative control yielded negative percentages; these were retained as measured for mean calculations, and negative mean values were truncated at zero for visualization only. Three independent BMV isolations were assayed per sample group (N = 3) with three technical replicates (n = 3).

### 2.15 Evaluation of immune-stimulatory effects via enzyme-linked immunosorbent assay

To assess the immune response elicited by BMVs, human monocytes (THP-1, passages 7-11, German Collection of Microorganisms and Cell Cultures GmbH, Braunschweig, Germany) were seeded at a density of 150,000 cells per well in a 24-well plate containing Roswell Park Memorial Institute (RPMI) medium (Thermo Fisher Scientific, Dreieich, Germany) supplemented with 10% FCS and 45 ng/mL phorbol 12-myristate 13-acetate to stimulate differentiation. After 48 h of incubation, cells were rested for an additional 24 h in fresh cell culture medium. Resting M0 macrophages were then exposed to BMVs at 10 µg/mL total protein for 12 h and 24 h (37°C, 5% CO_2_). Each BMV condition was run either with or without supplementation of polymyxin B (PMB; PHR1595, Merck KGaA, Darmstadt, Germany) at a final concentration of 30 µg/mL. BMVs were pre-incubated with PMB for 30 min at 37°C before addition to the cells. Controls comprised medium alone (RPMI/FCS), PMB alone (30 µg/mL), *P. aeruginosa* serotype 10.22-derived lipopolysaccharide (LPS) alone (L9143, Merck KGaA, Darmstadt, Germany) at a final concentration of 100 ng/mL, and LPS pre-incubated with PMB under identical conditions (30 min, 37°C). Afterward, supernatants were collected, centrifuged (13,000 × g, 4°C, 10 min), and stored at -80°C until further use. Concentrations of cytokines (IL-1β, IL-6, IL-10, TNF-α) were determined using uncoated ELISA kits for the respective proteins (Invitrogen, Waltham, USA) according to the manufacturer’s instructions. Absorbance was measured at 450 nm with reference correction at 570 nm (Tecan Spark, Männedorf, Switzerland). Experiments were performed with three independent BMV isolations per sample group, three independent cell passages (N = 3), and technical duplicates (n = 2).

### 2.16 Statistical analysis

Unless stated otherwise, N denotes the number of independent BMV isolations and independent cell passages (biological replicates); technical replicates (n) were averaged within each biological replicate before analysis. Single comparisons were assessed via unpaired two-tailed Student’s t-test, except for the CFU data, where the split-sample design was analyzed by a paired t-test. Group comparisons in MTT and LDH assays were performed using two-way ANOVA followed by Šídák’s multiple comparisons test in GraphPad Prism (v11.0.2, GraphPad Software, Boston, USA). Cytokine concentrations measured via ELISA were compared using two-tailed Welch unequal-variance t-tests, with p-values adjusted by Bonferroni correction applied within four pre-specified comparison families (LPS neutralization (8), planktonic versus biofilm BMVs (16), presence versus absence of polymyxin B (32), and BMV versus medium control (32); 88 comparisons in total). Cryo-TEM and nanoparticle tracking analysis were performed on combined preparations; the resulting data were not subjected to statistical analysis and are reported descriptively. Each figure legend states the number of biological and technical replicates, as well as the statistical test and correction method if applicable. Significance is indicated as * p < 0.05, ** p < 0.01 and *** p < 0.001, referring to adjusted p-values wherever a correction was applied.

## 3 Results

### 3.1 Isolation of bacterial membrane vesicles from planktonic and biofilm cultures

For this study, bacterial membrane vesicles (BMVs) were isolated from planktonic and biofilm cultures of *Pseudomonas aeruginosa* (*P. aeruginosa*) and *Staphylococcus aureus* (*S. aureus*) (**Figure 1A**). Since the biogenesis route of the vesicles analyzed here was not further elucidated, the term BMV was deliberately chosen for all membrane-enclosed particles released by the two organisms. Vesicles isolated from planktonic and biofilm cultures are abbreviated pBMVs and bBMVs, respectively. To ensure reliable comparability between the two culture approaches, the same planktonic pre-culture was used to inoculate both the planktonic culture and the fiber scaffold-based biofilms. The time point of BMV isolation was chosen based on growth behavior: planktonic cultures were harvested in the early stationary phase. Biofilm cultures were harvested after the scaffold (**Figure 1B**, blank scaffold) was fully colonized by bacteria and exhibited signs of biofilm development, including color changes and matrix formation. This time point was reached at 48 h for *P. aeruginosa* (**Figure 1C**) and at 72 h for *S. aureus* (**Figure 1D**). Full colonization of the scaffold was also confirmed via histology (**Supplementary Figure 1**). The biofilm matrix and bacteria were subsequently separated from the fiber scaffold in a bead beater and pelleted by centrifugation. For both sample groups, BMVs were isolated by ultracentrifugation.

**Figure 1:**
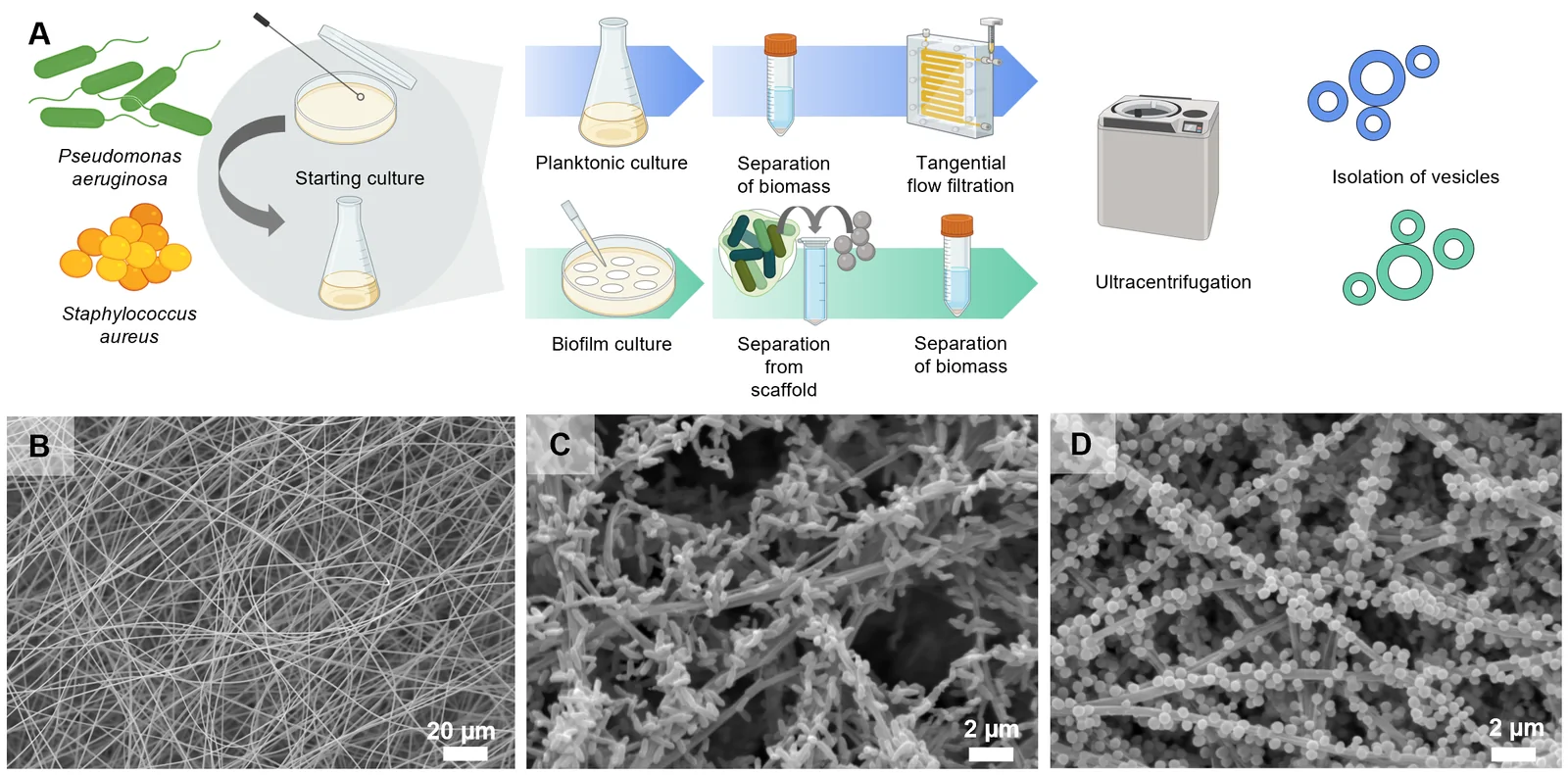
Schematic of experimental procedure and biofilm maturation. **(A)** Experimental procedure for bacterial membrane vesicle (BMV) isolation from planktonic and biofilm cultures of *P. aeruginosa* and *S. aureus*. Scanning electron micrographs of **(B)** electrospun fiber scaffold before inoculation, **(C)** the *P. aeruginosa* biofilm after 48 h, and **(D)** the *S. aureus* biofilm after 72 h of incubation.

To assess whether significant physical destruction occurred during isolation of bBMVs, bacterial viability was quantified after bead-beating. Thus, biofilms were processed as usual and colony-forming units (CFU) were determined (**Table 1**). *S. aureus* biofilms cultivated on the scaffolds reached a CFU/scaffold of 1.76 × 10^9^ (7.02 × 10^9^ CFU/mL), while *P. aeruginosa* was able to grow even denser, with a CFU/scaffold of 2.05 × 10^9^ (8.20 × 10^9^ CFU/mL). As CFU from non-disrupted biofilms could not be obtained, a split-sample design using planktonic bacteria was employed as a surrogate control for the disruption step. Three independent cultures of each organism were adjusted to an OD_600_ of 0.5 and divided into two aliquots, where one was subjected to the same bead-beating protocol used for biofilm disruption, and one was not further processed. Quantification of CFU highlighted that bead-beating did not significantly affect bacterial viability (**Table 1**). In the case of *S. aureus*, viable counts were slightly higher with a *Δ*log_10_ of 0.086 (8.653 ± 0.152 vs. 8.738 ± 0.094, p = 0.51), while a negligible decrease of *Δ*log_10_ 0.011 in colonies was observed for *P. aeruginosa* (8.995 ± 0.023 vs. 8.984 ± 0.039, p = 0.50).

**Table 1:** Viable bacterial counts before and after mechanical disruption of planktonic bacterial cultures, and within mature biofilms.

Planktonic cultures					
Organism	Beads	Mean (log_10_)	SD (log_10_)	CFU/mL	CFU/scaffold
*S. aureus*	–	8.653	0.152	4.49 × 10^8^	
	+	8.738	0.094	5.47 × 10^8^	
*P. aeruginosa*	–	8.995	0.023	9.88 × 10^8^	
	+	8.984	0.039	9.64 × 10^8^	
Biofilm					
Organism	Beads	Mean (log_10_)	SD (log_10_)	CFU/mL	CFU/scaffold
*S. aureus*	+	9.847	0.129	7.02 × 10^9^	1.76 × 10^9^
*P. aeruginosa*	+	9.914	0.407	8.20 × 10^9^	2.05 × 10^9^

Planktonic cultures of *P. aeruginosa* and *S. aureus* were adjusted to OD_600_ = 0.5 and split into one aliquot subjected to bead-beating and a parallel untreated aliquot. Colony-forming units (CFU) were determined by drop plating. Values are shown as the geometric mean ± SD of log_10_ CFU/mL from three independent samples per organism (N = 3), back-transformed to CFU/mL. Viable bacterial colonies in biofilm scaffolds could be assessed only after bead disruption and are shown as CFU/mL and CFU/scaffold.

### 3.2 Physicochemical differences of isolated BMVs

BMV samples were comprehensively analyzed for total protein content, hydrodynamic diameter, and zeta potential, as measures of BMV yield, size, and surface charge. Dynamic light scattering (DLS) resolved two distinct vesicle size populations in three of the four isolations, with one population below 50 nm and another ranging from 100 nm upward (**Figure 2A**). For *P. aeruginosa*, planktonic cultures produced vesicles with sizes of 36.1 ± 7.3 nm and 161.9 ± 31.4 nm, whereas biofilm-derived BMVs were measured at an average size of 157.3 ± 11.8 nm. *S. aureus* secreted BMVs of 33.5 ± 2.3 nm and 204.0 ± 88.4 nm when grown in a planktonic culture, and 33.8 ± 10.1 nm and 236.9 ± 38.9 nm when associated in biofilms; differences in BMV size were not significant (*P. aeruginosa* pBMVs vs. bBMVs: large population p = 0.796; *S. aureus* pBMVs vs. bBMVs: large population p = 0.521, small population p = 0.964). Polydispersity indices were 0.466 ± 0.068 and 0.268 ± 0.064 for *P. aeruginosa* BMVs from planktonic and biofilm cultures, and 0.547 ± 0.095 and 0.454 ± 0.041 for the corresponding *S. aureus* preparations (**Figure 2B**), highlighting the polydispersity of the different BMVs. The polydispersity index was significantly lower in *P. aeruginosa* bBMVs than in pBMVs (p = 0.0216), whereas the corresponding difference in *S. aureus* was not significant (p = 0.1922). The surface charge was negative in all preparations and more negative in bBMVs (**Figure 2C**). *P. aeruginosa* BMVs exhibited a surface charge of -8.8 ± 4.5 mV (pBMVs) and -13.2 ± 3.5 mV (bBMVs). For *S. aureus*, values of -8.5 ± 0.5 mV and -14.0 ± 1.9 mV were measured in planktonic and biofilm samples, respectively. The differences in surface charge were significant for *S. aureus* (p = 0.0014) but not for *P. aeruginosa* (p = 0.166). Additionally, size and surface charge were measured directly after isolation and after overnight storage at 4°C to assess potential storage-induced alterations; a size reduction and minor decreases in zeta potential were observed for *S. aureus* BMV populations (see **Supplementary Figure 2**). Cryo-transmission electron microscopy (cryo-TEM) of all four isolations confirmed the presence of intact, membrane-enclosed spherical particles (**Figure 3A**) but could not establish preparation purity, as smaller electron-dense material was also observed in the imaged fields. However, comparable structures were present in *S. aureus* (**Figures 3A**, left) and *P. aeruginosa* (**Figures 3A**, right) preparations from both growth modes, which also did not differ detectably in size. Grids prepared from single *S. aureus* isolations carried too few BMVs to be imaged reliably; thus cryo-TEM was performed after combining and further concentrating three biological replicates per sample group. Because intensity-weighted DLS distributions over-represent larger particles, nanoparticle tracking analysis (NTA) was conducted using the same combined preparations before concentration to provide number-weighted size distributions and particle concentrations for all four BMV isolates (**Figure 3B**); modal diameters of 113-133 nm and mean diameters of 148-211 nm (± 9 to ± 23 nm across the five captures) were measured. Particle concentrations in the dilutions were determined as 1.11 × 10^9^ mL^-1^ for *S. aureus* pBMVs, 9.96 × 10^8^ mL^-1^ for *S. aureus* bBMVs, 2.07 × 10^9^ mL^-1^ for *P. aeruginosa* pBMVs and 1.03 × 10^9^ mL^-1^ for *P. aeruginosa* bBMVs, corresponding to 4.44 × 10^9^, 3.98 × 10^9^, 4.14 × 10^9^ and 2.06 × 10^9^ particles/mL after correction for the dilution factor (**Supplementary Table 1**). Considering total protein amount, isolates from planktonic *P. aeruginosa* cultures yielded higher concentrations but showed greater variation per isolation (pBMVs 685.84 ± 109.11 µg/mL vs. bBMVs: 387.07 ± 32.01 µg/mL; p = 0.010). For *S. aureus*, total protein of pBMVs reached 999.62 ± 146.64 µg/mL, while BMVs isolated from biofilm cultures yielded a lower protein amount with a higher variability (728.96 ± 380.34 µg/mL; p = 0.314). Relating the particle concentrations measured via NTA to the total protein content of the same preparations resulted in particle-to-protein ratios of 4.44 × 10^6^, 5.46 × 10^6^ (*S. aureus* pBMVs and bBMVs), 6.04 × 10^6^, and 5.32 × 10^6^ (*P. aeruginosa* pBMVs and bBMVs) particles per µg protein, respectively (**Supplementary Table 1**). As the approximately 35 nm population falls below the practical detection limit of NTA, these ratios represent lower bounds rather than absolute rates and reproduced particle sizes measured via DLS for the larger BMV populations.

**Figure 2:**
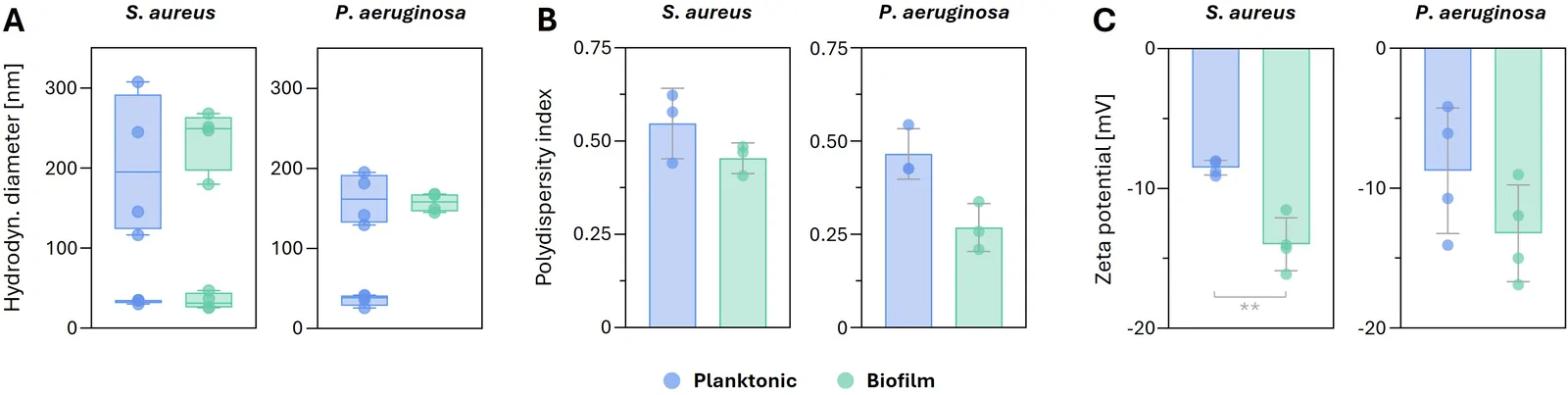
Physicochemical characterization of BMV preparations. **(A)** Intensity-weighted hydrodynamic diameter and **(B)** polydispersity indices measured by dynamic light scattering for planktonic (blue) and biofilm-derived (green) BMVs from *S. aureus* (left) and *P. aeruginosa* (right). **(C)** Zeta potential of the same preparations determined by electrophoretic light scattering. In **(A)**, boxes span the interquartile range, the center line marks the median, and whiskers mark the full range; **(B, C)** show mean ± SD; individual isolations are overlaid. N = 4 **(A, C)** and N = 3 **(B)**, each measured in triplicate (n = 3). Significance was determined by unpaired two-tailed Student’s t-test and is displayed as **p < 0.01.

**Figure 3:**
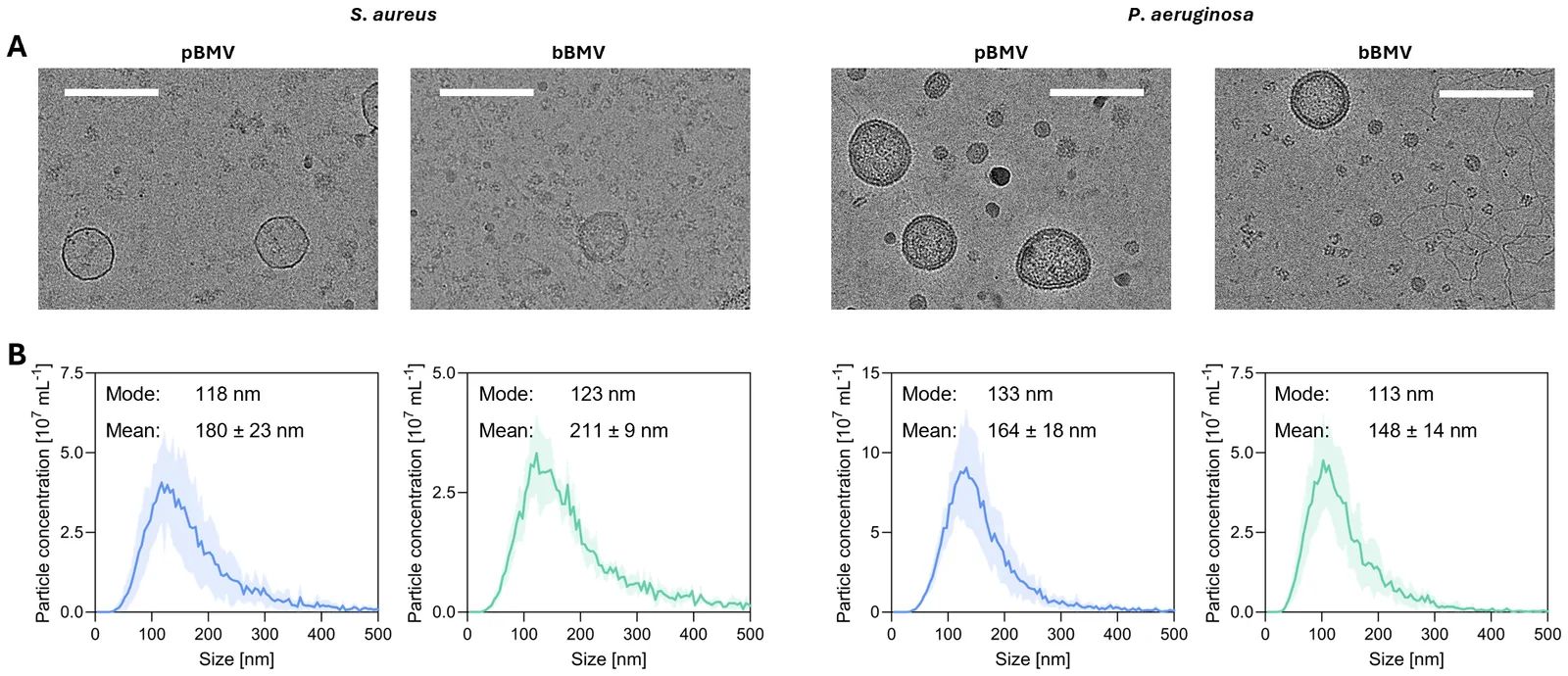
Vesicle identity and number-weighted size distribution. **(A)** Cryo-transmission electron micrographs (cryo-TEM) of pBMVs (blue) and bBMVs (green) from *S. aureus* (left) and *P. aeruginosa* cultures (right), showing intact spherical particles delimited by a single membrane bilayer. All four preparations showed comparable structures and no detectable size differences. Scale bars: 100 nm. **(B)** Number-weighted size distributions determined by nanoparticle tracking analysis (NTA) for the same four preparations. Distributions are binned at 5 nm (y-axis shows particle concentration within each bin; total particle concentrations are reported in **Supplementary Table 1**, diluted samples). Lines show the mean, and shaded bands show the SD across five captures per sample (technical replicates). Cryo-TEM and NTA were performed on preparations combining three independent biological replicates; no statistical comparison between the groups was conducted.

### 3.3 Growth mode reshapes the BMV proteome

To determine whether BMV protein composition also depended on culture conditions, label-free quantitative proteomics on three independent BMV isolations per sample group were performed. Filtering, imputation, normalization, and testing were carried out in Perseus; proteins were considered differentially abundant when the Benjamini-Hochberg-adjusted p-value was below 0.05, and the z-scored log_2_ intensity difference between growth modes was greater than 1 or less than -1. Differences are expressed as biofilm relative to planktonic throughout, so positive values denote enrichment in bBMVs. Complete values for all proteins, including adjusted p-values, z-scored intensity differences, peptide counts, and sequence coverage, are provided in **Supplementary Table 2**. Principal component analysis achieved clustering of pBMVs and bBMVs along the first principal component (PC1), accounting for 84.6% and 82.7% of the total variance in *S. aureus* and *P. aeruginosa*, respectively. While all three bBMV replicates clustered closely together, pBMV variation was higher along PC2 (**Supplementary Figure 3**).

A total of 930 proteins were identified in *S. aureus*-derived BMVs, of which 509 reached a Benjamini-Hochberg-adjusted p-value below 0.05 in the differential analysis; 259 of these also met the fold-change criteria, corresponding to 28% of all identified proteins: 121 were more abundant in bBMVs and 138 in pBMVs (**Figures 4A**, top). bBMVs were enriched in components of ATP synthesis and respiration (yellow; AtpA +1.08, AtpD +1.18, AtpE +2.92, AtpF +1.06, AtpH +1.21, Pyk +1.13, FumC +1.64, OdhA +1.64, OdhB +1.27; adj. p ≤ 0.01), in amino acid metabolism (blue; GcvT +1.73, GcvPB +1.12, GcvPA +1.06, ArgH +1.25, ArgG +2.34, ArgS +1.98, PurB +1.52; adj. p ≤ 0.018), in proteostasis (green; ClpP +1.34, Rny +1.39; adj. p ≤ 0.0026) and in surface proteins associated with biofilm physiology (red; PstS +1.13, PtsG +1.75, TarA +1.29, SrtA +1.19, SrrB +1.30, SpoVG +1.39; all adj. p ≤ 0.018). Sequence coverage for these proteins ranged from 17 to 94%, with the majority quantified from more than ten peptides. In contrast, *S. aureus* pBMVs (**Figures 4A**, bottom) carried more ribosomal subunits (yellow) and a pronounced set of secreted virulence factors (blue), including the staphylococcal superantigen-like protein Ssl10 (-1.87), the staphylococcal binder of immunoglobulins Sbi (-1.12), the hyaluronate lyase HysA (-1.94), the phospholipase Plc (-2.33), the polysaccharide intercellular adhesin deacetylase IcaB (-2.18), alpha-hemolysin Hly (-1.91), gamma-hemolysin subunits HlgB (-1.18) and HlgC (-1.77), leucotoxin LukDv (-1.82); all adj. p ≤ 0.015. The chaperone DnaK (-1.28) and the secreted antigen SsaA2 (-1.66), DNA repair and replication proteins (RpoE -1.46, ParC -1.59, PcrA -1.48, Mfd -1.54), further metabolic enzymes (FabZ -1.13, Fda -1.45, ValS -1.34), nucleotide and cofactor biosynthesis proteins (ThiI -1.43, PanB -1.60, ArgF -1.67) and cell wall biosynthesis proteins (FmtA -1.05, Emp -1.56, GlmU -2.09, VraR -1.87) were likewise more abundant in pBMVs (all adj. p ≤ 0.0139).

**Figure 4:**
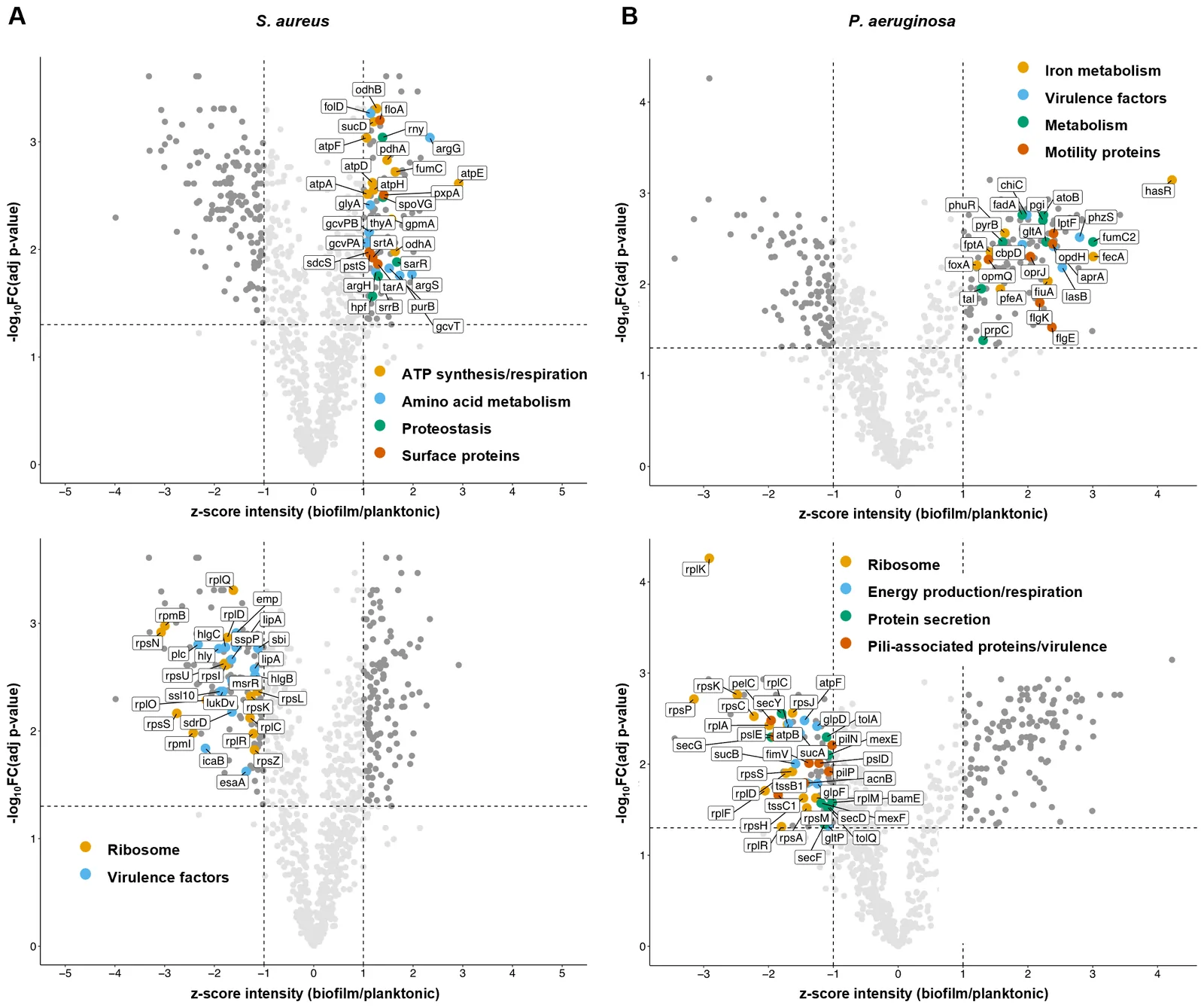
Quantitative proteome analysis of BMVs. Label-free quantitative proteomics of BMVs from **(A)** *S. aureus* and **(B)** *P. aeruginosa* grown as planktonic or biofilm cultures, shown as volcano plots of z-scored intensity (biofilm relative to planktonic; positive values denote enrichment in bBMVs) against the Benjamini-Hochberg-adjusted p-value. Proteins were considered differentially abundant at adjusted p < 0.05 and a difference in z-scored log_2_ intensity >1 or <-1 (dashed lines). Data are derived from three independent BMV isolations per sample group (N = 3). Of 930 proteins quantified in *S. aureus* BMVs, 259 met the enrichment criteria; 121 were enriched in bBMVs and 138 in pBMVs. Of 793 proteins quantified in *P. aeruginosa* BMVs, 230 proteins met the enrichment criteria; 135 were enriched in bBMVs and 95 in pBMVs. Values for all proteins, including adjusted p-values, fold changes, peptide counts, and sequence coverage, are supplemented in **Supplementary Table 2**.

In the case of *P. aeruginosa*-derived BMVs, a total of 793 proteins were identified, 383 reached a Benjamini-Hochberg-adjusted p-value below 0.05 in the differential analysis, and 230 of these further met the fold-change criteria, corresponding to 29% of all identified proteins. Out of these, 135 were more abundant in bBMVs and 95 in pBMVs (**Figure 4B**; full data set in **Supplementary Table 2**). bBMVs (**Figures 4B**, top) were most strongly enriched in heme acquisition systems (yellow): heme uptake receptor HasR (+4.22, adj. p = 7.2 × 10^-4^), ferric citrate transporter FecA (+3.01; adj. p = 0.005) and heme receptor PhuR (+1.65; adj. p = 0.0027), together with the pyoverdine biosynthesis proteins PvdN (+2.32; adj. p = 0.0028) and PvdO (+1.61, adj. p = 0.0043) and the pyochelin receptor FptA (+1.43; adj. p = 0.0045). Secreted virulence factors (blue) were enriched in the same fraction (elastase LasB +2.53, alkaline protease AprA +2.39, chitin-binding protein CbpD +1.91, chitinase ChiC +1.98; all adj. p ≤ 0.0065), as were proteins involved in general carbon and energy metabolism (green), such as the TCA cycle enzymes fumarate hydratase FumC2 (+3.00) and citrate synthase GltA (+2.28), acetyl-CoA acetyltransferase AtoB (+2.24), glucose-6-phosphate isomerase Pgi (+2.23), transaldolase Tal (+1.28) and the methylcitrate pathway enzyme 2-methylcitrate dehydratase PrpD (+2.34) (all adj. p ≤ 0.012). Flagellar structural proteins (red) were also more abundant in this fraction (the hook protein FlgE +2.37 and the hook-associated protein FlgK +2.18; adj. p ≤ 0.0295). Further enriched in bBMVs were proteins involved in oxidative stress defenses (catalase KatA +2.78, superoxide dismutase SodB +2.47, thioredoxin peroxidase Tpx +2.14, adj. p ≤ 0.0066), and proteins associated with antibiotic resistance (efflux porin OprJ +2.04 and AmpD homolog AmpDh3 + 2.55; adj. p = 0.0058). The phenazine-modifying enzyme PhzS, which catalyzes the terminal step of pyocyanin biosynthesis, was likewise enriched in bBMVs (+2.80, adj. p = 3.0 × 10^-^³). *P. aeruginosa* pBMVs (**Figures 4B**, bottom) contained higher levels of proteins involved in ribosomal protein synthesis (yellow), which account for the largest differences (RpsP -3.15, RpmD -2.92, RplK -2.91, RpsK -2.48, RpsC -2.22; adj. p ≤ 0.003), together with components of ATP synthesis and respiration (blue; AtpB -1.69, AtpF -1.45, GlpD -1.25, NuoA2 -1.18; adj. p ≤ 0.004), and the Sec protein export machinery (green; SecG -1.96, SecY -1.80, SecF -1.14, SecD -1.06; adj. p ≤ 0.046). Type IV pilus and motility-associated proteins were also more abundant in pBMVs, including the polar motility hub FimV (-1.38) and the pilus inner membrane components PilP (-1.07) and PilN (-1.02) (adj. p ≤ 0.012). Additionally, proteins involved in protein folding and stress response (RecA -1.27, IbpA -1.07, DnaJ -1.02, HtpG -1.01; adj. p ≤ 0.044), components of the Type VI secretion system (TssC1 -1.85, TssB1 -1.44, Hcp1 -1.28; adj. p ≤ 0.023) and the multidrug efflux components MexF (-1.19) and MexE (-1.08) (adj. p ≤ 0.027) were also found to be significantly more abundant in pBMVs. Notably, the exopolysaccharide biosynthesis proteins of the Psl and Pel systems were enriched in planktonic- rather than biofilm-derived vesicles (PslE -1.90, PelC -1.96, PslD -1.22; all adj. p ≤ 0.010), with only the alginate regulatory protein AlgP more abundant in biofilm-derived vesicles (+1.13). Matrix biosynthesis determinants are therefore not preferentially represented in vesicles released by biofilm cultures. Proteins that were not differentially abundant between growth conditions were predominantly associated with general bacterial survival and cell maintenance.

### 3.4 BMVs reduce epithelial viability with limited membrane rupture

BMVs were further characterized concerning their cytotoxic potential using two human epithelial cell lines and two orthogonal assays. Caco-2 enterocytes and HaCaT keratinocytes were exposed to BMVs at 10, 25, 50, and 100 µg/mL total protein for 24 h, and metabolic activity (MTT, **Figure 5**) and plasma membrane integrity (LDH release, **Figure 6**) were measured in parallel and compared with a buffer-treated control. Concerning metabolic activity, treatment of Caco-2 cells with *S. aureus*-derived BMVs resulted in a minor reduction for both BMV sample groups (bBMVs 101.2 ± 3.9% to 77.9 ± 10.2%; pBMVs 94.0 ± 4.8% to 86.1 ± 16.3%) (**Figures 5A**, left). *P. aeruginosa* bBMVs reduced metabolic activity in a concentration-dependent manner from 77.3 ± 4.2% at 10 µg/mL to 65.0 ± 11.0% at 100 µg/mL, compared with 87.7 ± 5.8% and 84.1 ± 6.8% for pBMVs (**Figures 5A**, right). In the case of BMVs isolated from *P. aeruginosa*, HaCaT keratinocytes reacted more sensitively than Caco-2 cells (**Figure 5B**). *P. aeruginosa* bBMVs reduced metabolic activity to 48.7 ± 4.4% already at 10 µg/mL and to 41.7 ± 5.7% at 100 µg/mL, against 83.4 ± 8.4% and 60.2 ± 7.4% for pBMVs. *S. aureus* BMVs had only a limited effect on this cell line, with metabolic activity remaining between 82.2 ± 12.0% (10 µg/mL) and 77.4 ± 15.9% (100 µg/mL) for bBMVs and between 80.1 ± 2.8% and 81.3 ± 10.5% for pBMVs, without a clear concentration dependence. While a higher cytotoxic effect of *P. aeruginosa*- relative to *S. aureus*-derived BMVs was reproduced in both cell lines, differences in biofilm and planktonic growth were only consistent for *P. aeruginosa*, where the difference between growth modes was significant in Caco-2 cells at 50 and 100 µg/mL (adj. p = 0.020 and adj. p = 0.0093) and in HaCaT cells at all four concentrations (adj. p = 0.0001, 0.012, 0.010 and 0.030). None of the *S. aureus* comparisons reached significance. LDH release remained modest across all conditions and, unlike the MTT readout, increased with vesicle concentration in nearly every series (**Figure 6**). In Caco-2 cells, release stayed below 11% up to 50 µg/mL for all four preparations and rose only at 100 µg/mL, reaching a maximum of 21.2 ± 10.0% for *P. aeruginosa* pBMVs and 18.8 ± 11.0% for *S. aureus* bBMVs (**Figure 6A**). HaCaT cells behaved similarly, with values between 0.8 ± 6.4% and 14.1 ± 4.5% up to 50 µg/mL and a maximum of 28.0 ± 1.4% for *S. aureus* bBMVs at 100 µg/mL (**Figure 6B**). Despite high standard deviation, two pairwise comparisons reached significance after Šídák correction: treatment with *P. aeruginosa* pBMVs at 100 µg/mL resulted in a higher release of LDH than bBMVs in Caco-2 cells (adj. p = 0.019), and application of *S. aureus* bBMVs led to a higher release compared to pBMVs at this concentration in HaCaT cells (adj. p = 0.035); all remaining comparisons were non-significant.

**Figure 5:**
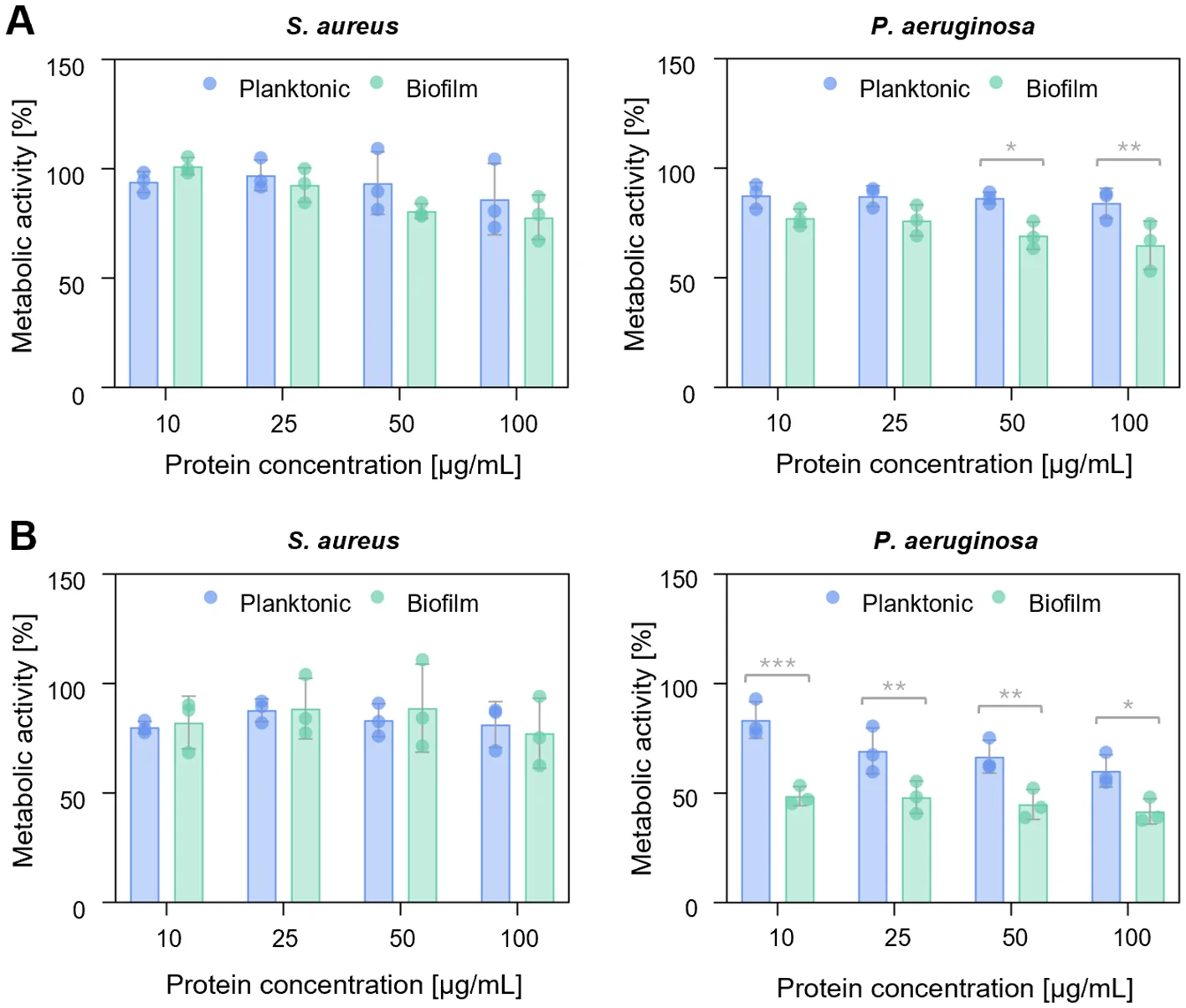
Effect of BMVs on epithelial metabolic activity. **(A)** Caco-2 enterocytes and **(B)** HaCaT keratinocytes were exposed to BMVs from *S. aureus* (left) and *P. aeruginosa* (right) at 10, 25, 50, and 100 µg/mL total protein, isolated from planktonic (blue) or biofilm (green) cultures. Metabolic activity was measured by MTT assay after 24 h incubation and is expressed as a percentage of the buffer-treated control. Data are depicted as mean ± SD of N = 3 independent BMV isolations, each measured in three technical replicates (n = 3). Group comparisons were made by two-way ANOVA followed by Šídák’s multiple comparisons test, and statistical significance is indicated as * p < 0.05, ** p < 0.01, and *** p < 0.001.

**Figure 6:**
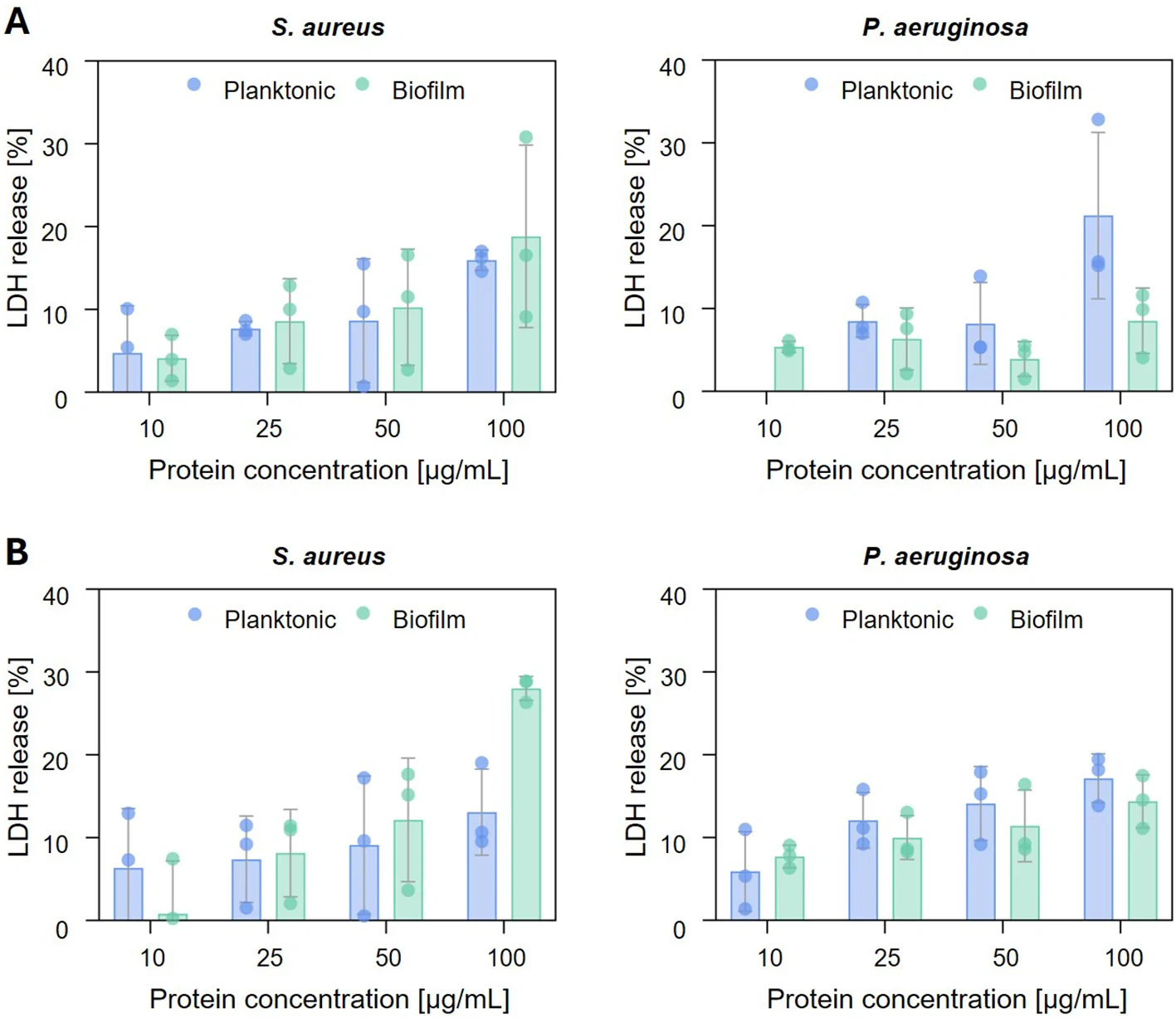
Effect of BMVs on epithelial plasma membrane integrity. **(A)** Caco-2 enterocytes and **(B)** HaCaT keratinocytes were treated with BMVs from *S. aureus* (left) and *P. aeruginosa* (right) at 10, 25, 50, and 100 µg/mL total protein, isolated from planktonic (blue) or biofilm (green) cultures. Lactate dehydrogenase (LDH) released into the supernatant was quantified after 24 h and is expressed as a percentage of the maximal LDH release (1% Triton X-100 in culture medium); PBS-treated cells served as the negative control. Data are depicted as mean ± SD of N = 3 independent BMV isolations, each measured in three technical replicates (n = 3). Statistical group comparisons were made by two-way ANOVA followed by Šídák’s multiple comparisons test. Values falling below the spontaneous LDH release of the negative control yielded negative percentages and were retained for mean calculation, and only truncated at zero for visualization.

### 3.5 BMVs elicit a cytokine response that is not solely attributable to free endotoxin

For the evaluation of immune stimulatory effects, human M0 macrophages were exposed to BMVs at 10 µg/mL total protein, and IL-1β, IL-10, IL-6, and TNF-α were quantified in the supernatant after 12 h and 24 h via ELISA (N = 3, **Figures 7A–D**). Each condition was run in parallel with and without polymyxin B (PMB, 30 µg/mL). Data are reported as absolute concentrations, since the medium baseline changed between time points and fold-change expression would confound that shift with the treatment effect. Assay controls performed as intended: PMB neutralized 45-77% of the free-LPS response at 12 h and 78-95% at 24 h, which reached significance after correction only for IL-6 at 24 h (adj. p = 0.027) (**Supplementary Figure 4**).

**Figure 7:**
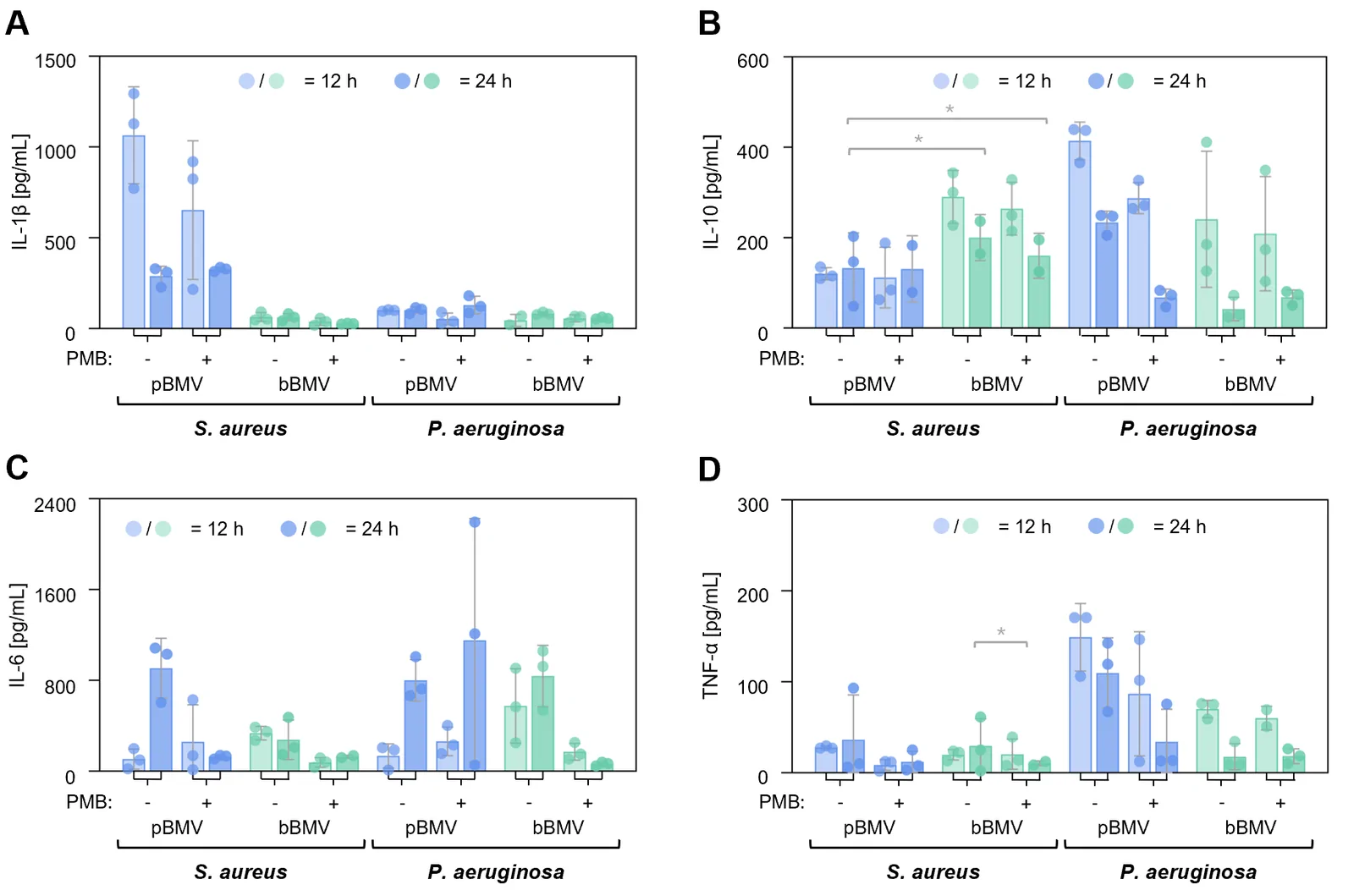
Cytokine response of human macrophages to BMVs. THP-1-derived M0 macrophages were exposed for 12 h and 24 h to BMVs at 10 µg/mL total protein from planktonic or biofilm cultures of *S. aureus* and *P. aeruginosa*, each in the absence (-) and presence (+) of polymyxin B (PMB, 30 µg/mL). Concentrations of **(A)** IL-1β, **(B)** IL-10, **(C)** IL-6, and **(D)** TNF-α in the culture supernatant were quantified by ELISA and are reported as absolute concentrations (pg/mL). Medium-treated (RPMI) and LPS-treated (LPS, end concentration 100 ng/mL) cells served as negative and positive controls, respectively. Further controls included sole PMB treatment (30 µg/mL) and neutralization control (LPS + PMB) (see **Supplementary Figure 4**). Data are plotted as mean ± SD (N = 3, n = 2). Statistics were carried out using a two-tailed Welch unequal-variance t-test with Bonferroni correction. *p < 0.05 after correction.

IL-1β release was highest overall in response to *S. aureus* pBMVs (1064.8 ± 266.5 pg/mL at 12 h), declining to 289.3 ± 54.0 pg/mL by 24 h. *S. aureus* bBMVs induced substantially less IL-1β secretion (63.7 ± 24.2 pg/mL at 12 h; 62.2 ± 19.4 pg/mL at 24 h). IL-1β release by *P. aeruginosa*-derived BMVs did not prominently differ across growth modes (pBMVs: 100.6 ± 4.4 and 101.4 ± 17.4 pg/mL; bBMVs: 45.1 ± 33.7 pg/mL and 82.2 ± 7.2 pg/mL, at 12 and 24 h, respectively) (**Figure 7A**). Growth-mode dependence was most pronounced for IL-10 (**Figure 7B**). Treatment with *P. aeruginosa* pBMVs induced a release of 414.0 ± 42.0 pg/mL at 12 h and 233.8 ± 25.0 pg/mL at 24 h, compared with 240.9 ± 150.6 and 42.2 ± 26.0 pg/mL for bBMVs; the difference was significant after correction (adj. p = 0.012). For *S. aureus*, the pattern was reversed, with application of bBMVs eliciting higher IL-10 secretion than pBMVs (290.2 ± 58.8 vs. 120.1 ± 13.6 pg/mL at 12 h), although with no statistical significance. IL-6 kinetics differed between planktonic and biofilm-derived BMVs (**Figure 7C**). pBMVs from both species elicited IL-6 release that continued to increase between 12 and 24 h (*S. aureus*: 106.1 ± 89.4 to 907.3 ± 263.1 pg/mL; *P. aeruginosa*: 134.3 ± 107.6 to 800.2 ± 183.0 pg/mL). Treatment with bBMV, instead, resulted in IL-6 responses that were already elevated at 12 h (*S. aureus*: 335.0 ± 59.5 pg/mL, decreasing to 276.9 ± 174.8 pg/mL by 24 h; *P. aeruginosa*: 575.2 ± 328.1 pg/mL, increasing further to 838.8 ± 271.1 pg/mL). TNF-α release was highest after application of *P. aeruginosa* pBMVs (149.0 ± 37.2 pg/mL at 12 h) (**Figure 7D**). PMB co-treatment did not completely suppress vesicle-induced cytokine release under conditions in which it substantially reduced the macrophage response to free LPS. For *P. aeruginosa* pBMVs at 24 h, PMB co-treatment even increased IL-1β by 28% (101.4 to 129.6 pg/mL) and IL-6 by 44% (800.2 to 1153.3 pg/mL), although with very high sample standard deviations. PMB also reduced *S. aureus* BMV responses by up to 86% despite the lack of LPS in this Gram-positive organism, although none of these reductions reached significance after correction.

## 4 Discussion

Bacterial membrane vesicles (BMVs) have gained increasing recognition as key mediators of intercellular communication, virulence, and host-pathogen interaction. Yet, how BMV composition and function are shaped by bacterial growth modes remains poorly defined. By systematically comparing BMVs from *S. aureus* and *P. aeruginosa* across both growth states, this study demonstrates that growth conditions profoundly and functionally influence vesicle composition and biological activity, with important implications for understanding infection pathogenesis.

To mimic biofilm growth in a human tissue environment, a previously established model based on electrospun fibers emulating the extracellular matrix was used (Wächter et al., 2023). Biofilms were harvested at full maturity, defined as complete scaffold colonization with visible matrix formation, which was reached at 72 h for *S. aureus* and 48 h for *P. aeruginosa*; at harvest, the biofilms carried between 7.0-8.2 × 10^9^ CFU/mL. Planktonic cultures were cultivated until the transition from exponential to stationary phase, corresponding to the most densely inhabited phase. Controlling and standardizing bacterial growth in this way limits potential interference from differing life states or stressors, such as starvation, leaving growth mode and species as the principal variables distinguishing the four preparations.

Physicochemical characterization confirmed that BMV diameters fell within the range previously reported for bacterial vesicles, with *S. aureus* BMVs generally larger than *P. aeruginosa* BMVs (Bauman and Kuehn, 2006; Bose et al., 2021; Brown et al., 2015). Interestingly, two distinct vesicular populations were observed in all isolated samples, except in *P. aeruginosa* biofilm-derived BMVs, consistent with prior reports linking multiple size populations to differences in cargo and function (Cooke et al., 2019; Johnston et al., 2023). No statistically significant growth-dependent changes were observed, consistent with current evidence, suggesting that vesicle diameter is more strongly influenced by strain identity than by growth state (Cooke et al., 2019). Cryo-TEM and number-weighted NTA were conducted on combined samples. Although data interpretation is limited to the composite of three individual BMV isolations rather than determining replicate-based variations, and observations are purely descriptive, micrographs showed intact, membrane-enclosed vesicles in all four BMV preparations. Additionally, NTA supported DLS-based measurements, reproducing the size measurements for the larger BMV population. The smaller population with a vesicle diameter of approximately 35 nm fell below the practical detection limit of this technique (Filipe et al., 2010). Values obtained from these preparations describe the material measured and were not used to compare vesicle output between growth modes. In contrast, zeta potential measurements revealed a consistently more negative surface charge in biofilm-derived BMVs across both species (approx. -9 mV vs. -14 mV). While both values were consistent with the usual surface charges of bacterial vesicles, the significant shift in biofilm BMVs might indicate molecular differences in charged surface-exposed moieties, such as carboxylic groups (Rogers et al., 2023). For example, adaptations in membrane components that contribute to surface charge, such as lipopolysaccharides, have been identified in *P. aeruginosa* cells across different growth conditions, suggesting subsequent alterations in BMVs (Lima et al., 2019).

Assessing the proteome of BMVs from both planktonic and biofilm-associated cultures revealed differential abundance of certain protein families depending on BMV origin. Most notably, across both *S. aureus* and *P. aeruginosa*, planktonic-derived vesicles carried enriched amounts of ribosomal proteins, indicating an active state of protein synthesis and rapid cell growth (Aseev et al., 2024; Cheng-Guang and Gualerzi, 2021; Laursen et al., 2005); in *P. aeruginosa*, these account for the largest negative difference in the dataset. This is consistent with planktonic cells actively proliferating at the time of BMV isolation, requiring a robust translation machinery. Furthermore, virulence factors were particularly abundant in BMVs from planktonic cultures, including toxins and enzymes that degrade host tissues. These proteins suggest that planktonic cells were primed for acute infection, which requires rapid growth and virulence factor production (Di Bella et al., 2025; Liao et al., 2022; Qin et al., 2022). Additionally, proteins associated with membrane transporters, especially those relevant to nutrient uptake, such as sugar or amino acids, were enriched in pBMVs (Davies et al., 2021; Malviya et al., 2023; Zeden et al., 2020); the enrichment of nutrient-uptake proteins is consistent with planktonic cells actively scavenging during rapid growth. Despite their active growth, the cells also enriched stress-response proteins, including chaperones, which help manage stress associated with fast cell division and potentially hostile environments (Ghazaei, 2017; Miwa et al., 2021). In *S. aureus*, this fraction comprises the chaperone DnaK together with a set of DNA repair and replication proteins. Notably, this arm of the stress response was enriched in pBMVs and not in bBMVs, the opposite of the pattern usually assumed for biofilms. However, this study did not evaluate bacterial growth kinetics, metabolic flux, or transcriptional state, which may also influence BMV loading of cellular stress signals and modulators.

While variations between *S. aureus* and *P. aeruginosa* were not that prominent for planktonic BMVs, the proteome of biofilm-derived BMVs crucially differed between the two bacterial species. In *S. aureus* bBMVs, the proteome included numerous enzymes involved in various metabolic pathways, along with ATP synthase subunits, indicating adaptation to nutrient-limited conditions common in biofilms. The presence of enzymes from glycolysis, the tricarboxylic acid cycle, and amino acid biosynthesis suggests that biofilms were metabolically active and adapted to the nutrient conditions of a mature biofilm rather than metabolically dormant (Malviya et al., 2023; Pisithkul et al., 2019). Surface and envelope proteins associated with biofilm physiology were also enriched, specifically the wall teichoic acid biosynthesis protein TarA, sortase A, and the phosphate-binding protein PstS and the PTS component PtsG (Malviya et al., 2023; Pisithkul et al., 2019). Those proteins are crucial to the stability and integrity of the biofilm and are essential for its survival and persistence in hostile environments (Rahman et al., 2022; Speziale et al., 2014). Other cell wall biosynthesis proteins, such as FmtA, GlmU, or VraR, were more abundant in pBMVs, suggesting that proteins associated with envelope biosynthesis are preferentially packaged into pBMVs.

Stress-response proteins might have been expected to be enriched in biofilm-derived vesicles, reflecting the adaptive mechanisms that *S. aureus* employs to survive in the challenging biofilm environment. Such proteins, involved in oxidative stress response, protein folding, and repair systems, help bacteria resist external stressors such as host immune responses and antibiotic treatments (Ghazaei, 2017; Ha and Edwards, 2021). However, neither catalase, superoxide dismutase, nor alkyl hydroperoxide reductase were found to be differentially abundant in *S. aureus*-derived bBMVs, and the chaperone and DNA repair proteins were additionally enriched in pBMVs instead. Other enriched functional categories encompassed membrane transport systems, permeability, signaling, and chemotaxis (Costa et al., 2023; Gélinas et al., 2021; Peng et al., 2022). In addition to these growth-influenced shifts, many detected proteins were not significantly enriched in either state. Most non-differentially abundant proteins are known to perform basic cellular functions and contribute significantly to bacterial survival in both planktonic and biofilm cultures (Briaud et al., 2021; Lee et al., 2009; Tartaglia et al., 2020; Uppu et al., 2023).

pBMVs of *P. aeruginosa* shared most of the enriched protein classes found in *S. aureus*. Motility-associated proteins, however, were split between the two growth modes. Type IV pilus and motility components were more abundant in planktonic-derived vesicles (FimV, PilP, PilN), consistent with the motility that planktonic *P. aeruginosa* cells require for colonization, invasion, and dispersal (Rico-Jiménez et al., 2013; Talà et al., 2019; Wei et al., 2009), whereas the flagellar hook proteins FlgE and FlgK were enriched in bBMVs. In contrast, proteins mediating iron acquisition and storage, such as siderophores and their receptors, were significantly enriched in bBMVs and contributed the largest positive differences in the entire dataset (HasR, FecA and PhuR, together with the pyoverdine biosynthesis proteins PvdN and PvdO and the pyochelin receptor FptA). These proteins help *P. aeruginosa* sequester iron from the environment, supporting biofilm growth and maintenance and potentially counteracting iron deficiencies, as this element is often limited in the biofilm microenvironment (Zhang et al., 2021). Matrix biosynthesis determinants, by contrast, were not preferentially packaged into bBMVs, e. g., the Psl and Pel exopolysaccharide proteins were more abundant in planktonic-derived vesicles, with only the alginate regulatory protein AlgP enriched in the biofilm fraction (Tseng et al., 2018; Zhang et al., 2015). An equivalent observation was made for *S. aureus*, as the polysaccharide intercellular adhesin deacetylase IcaB was likewise enriched in pBMVs. No component of the Las, Rhl, or Pqs quorum sensing systems was differentially abundant in either direction. Another major group of proteins, predominantly present in *P. aeruginosa* bBMVs, included virulence factors and other proteins that shape host-cell interactions, promoting immune evasion and antibiotic resistance, such as elastase LasB, alkaline protease AprA, and the oxidative stress defense proteins KatA and SodB. The presence of these molecules, including extracellular enzymes and toxins, suggests that biofilm vesicles may contribute to pathogenesis by promoting tissue damage and immune evasion during chronic infections, consistent with prior findings (Couto et al., 2015; Park et al., 2014).

The proteomic profiles of bacterial vesicles represent a highly selective subset of the total bacterial proteome, reflecting both the physiological state of the parent cell and active cargo-sorting processes rather than passive membrane shedding (Juodeikis and Carding, 2022; Schwechheimer and Kuehn, 2015). Mechanical disruption during sample preparation was analyzed as a potential source of the cytoplasmic and ribosomal proteins measured in this study. Because viable cell counts of non-disrupted biofilms cannot be quantified, planktonic cultures were used as a surrogate. Subjection of bacterial cells to bead-beating did not significantly alter the number of colony-forming units in either tested organism. Gross lysis during sample preparation is therefore an unlikely explanation for the significant enrichment of cytoplasmic proteins. CFU, however, assesses viability only, leaving sub-lethal membrane damage undetected, and biofilm-embedded cells may be more or less susceptible to bead-beating than cells in a planktonic culture. To exclude the entrance or attachment of highly abundant cytoplasmic proteins into or to BMVs through routes other than regulated sorting mechanisms, paired vesicle and whole-cell proteome comparisons would be needed for further interpretation. However, these findings support the current literature and highlight the complexity of bacterial vesicle packaging, which does not simply appear to be a proxy for the parent cell’s composition.

To evaluate the functional consequences of these compositional differences, the cytotoxic and immunogenic potential of BMVs was assessed in human cell models. Dosing was normalized to total protein amount rather than particle number; thus, cytotoxicity and cytokine readouts between preparations compare equal protein loads, not equal vesicle numbers. *P. aeruginosa* BMVs induced concentration-dependent reductions in metabolic activity and were generally more cytotoxic than *S. aureus* BMVs in both Caco-2 enterocytes and HaCaT keratinocytes. Further, HaCaT cells were more sensitive to *P. aeruginosa* BMVs, and biofilm-derived vesicles of this organism were also more cytotoxic than planktonic isolations. Metabolic activity fell to as little as 41.7%, LDH release never exceeded 28% and remained below 11% up to 50 µg/mL in Caco-2 cells. For *P. aeruginosa* and Caco-2 cells, bBMVs reduced metabolic activity more than pBMVs, yet the highest LDH release recorded in that cell line came from the planktonic preparation. These findings therefore suggest that suppression of metabolic activity is the dominant effect of BMV exposure in these cell lines, rather than plasma membrane lysis. Additionally, bBMVs and pBMVs appear to act through different modes: while the former seem to be comparatively more metabolically suppressive, pBMVs might act more membranolytically in Caco-2 cells, although the only significant LDH difference, seen in HaCaT cells, ran in the opposite direction. For *P. aeruginosa*, bBMVs significantly reduced metabolic activity in both cell lines; the remaining observations describe consistent trends that require further evaluation.

Secretion of the key pro-inflammatory cytokines IL-1β, IL-6, and TNF-α, as well as the anti-inflammatory cytokine IL-10, was monitored in M0 macrophage monocultures following a 12 h and 24 h BMV treatment. All four preparations were applied at the same total protein concentration, so these differences reflect what the vesicles carry rather than how much vesicle material the cells received. Species- and growth mode-dependent differences in cytokine responses were observed, including secreted amount and secretion onset. *S. aureus* BMVs particularly increased IL-1β secretion, potentially through TLR-2 priming, NLRP3 inflammasome activation, and pore-forming toxins, thus leading to robust IL-1β and IL-18 release and pyroptosis (Wang et al., 2020). pBMVs, which were enriched in the hemolysins and leucocidins Hly, HlgB, HlgC, and LukDv, induced the highest secretion over all conditions analyzed. bBMVs, which carried less of these toxins, elicited a markedly lower response, although this difference did not reach significance after correction. Additionally, for *S. aureus*, none of its highly potent toxins, such as α-hemolysin, appear specifically enriched in bBMVs (Di Bella et al., 2025).

Treatment with *P. aeruginosa* BMVs, in contrast, resulted in the highest TNF-α response out of the four BMV preparations tested, which is in line with previous reports (Ellis et al., 2010). Further, influences of growth modes were observed in the anti-inflammatory IL-10 arm. Treatment with bBMVs resulted in reduced IL-10 secretion compared to pBMVs at both time points. Given the earlier onset of IL-6 and the greater loss of epithelial viability, this BMV preparation is the least likely to trigger cellular counter-regulation. Potential contributors to this behavior are toxins and virulence factors enriched in bBMVs, such as the prominent metalloprotease elastase (LasB), which was highly enriched in bBMVs (Cheung et al., 2014; Di Bella et al., 2025; Liao et al., 2022). This enzyme degrades extracellular matrix proteins, disrupts cell junctions, and induces the secretion of pro-inflammatory cytokines. Further, the phenazine-modifying enzyme PhzS catalyzes the terminal step in the synthesis of pyocyanin, a redox-active toxin that imposes oxidative stress on host cells (Qin et al., 2022).

The analyzed cytokines represent temporally distinct phases of the inflammatory response. TNF-α is released rapidly, IL-1β accumulates more slowly and is sustained over time, and IL-6 kinetics fall between the two (Medzhitov and Janeway, 2000). Growth mode affected the timing of the response as much as its magnitude, especially highlighted by the convergence in IL-6 induction. pBMV isolations of both species produced IL-6 responses that were still climbing at 24 h, whereas bBMV-driven secretion was already substantially elevated at 12 h, suggesting that bBMVs engage comparatively early with macrophages. Notably, pBMVs induced stronger IL-1β and TNF-α secretion despite biofilm-derived BMVs exhibiting greater cytotoxicity, whereas IL-6 was higher in response to bBMVs at 12 h. This dissociation may point to active immune suppression by bBMVs. These data cannot directly resolve whether this reflects differences in uptake rate, ligand density, or the ligands themselves. Meanwhile, the IL-10 difference diverged between species, highlighting differences in the imposed immunological signature.

In an approach to exclude potential confounding effects of LPS in BMVs secreted by the Gram-negative *P. aeruginosa*, macrophages were also treated with BMVs pre-incubated with polymyxin B (PMB). Controls showed that the applied PMB concentration neutralized 45-77% of the free-LPS response at 12 h and 78-95% at 24 h, yet did not completely abolish the vesicle-induced response. Interestingly, PMB addition even increased cytokine responses to *P. aeruginosa* pBMVs for IL-1β and IL-6, although with very high standard deviations, which may be explained by incomplete neutralization of vesicle-bound LPS and subsequent rapid uptake and delivery of BMVs into endosomes, where LPS is not accessible to PMB (Vanaja et al., 2016). Moreover, PMB also reduced cytokine secretion induced by *S. aureus* BMVs, which are devoid of LPS, a result attributed to potential affinity for lipoteichoic acid or other bacterial ligands present in Gram-positive bacteria (Heydarian et al., 2024). Thus, further experiments, including direct quantification of endotoxins and other virulence factors, are needed to clearly distinguish their relative contribution to BMV-induced cytokine responses.

This study provides a systematic cross-species comparison of proteinaceous BMV cargo and host response using reference strains (*S. aureus* ATCC 29213, *P. aeruginosa* ATCC 27853), selected to maximize comparability and reproducibility for an initial comparison of this kind. As vesicle production, biofilm architecture, and virulence factor expression vary among different isolates, these findings establish species- and strain-specific baselines; extending the analysis to clinical and multidrug-resistant isolates is a priority for future work. Further, with three biological replicates per condition, the study is powered to detect large, consistent effects. Replicate-level quality control supports the internal consistency of the proteomic datasets, as replicates of the same growth mode grouped together in principal component analysis and correlated more closely with one another than with the opposite growth mode (**Supplementary Figure 3**), indicating that growth mode was the dominant source of variation in both datasets. Increasing the number of replicates in follow-up experiments will further confirm the trends observed in this study. Future experiments should also pair cryo-TEM with a co-isolate-specific marker to directly quantify preparation purity. Further, establishing whether particle-to-protein ratios and particle concentrations differ systematically between growth modes would require NTA on individual biological replicates, and remains an objective for future work. Direct endotoxin quantification, alongside orthogonal PMB neutralization approaches, is needed to interpret the immune-modulatory effects elicited by different isolations. Although BMV cargo and immunological responses were consistent, proteome analysis cannot resolve all factors that may contribute to downstream cellular effects. For example, small phenol-soluble modulins, consisting only of 20 to 30 amino acids, are routinely missed by broad proteomic analyses and require specialized approaches (Cheung et al., 2014). Attributing the cytokine and cytotoxicity results to individual constituents would require toxin quantification, inhibition assays, or mutant analyses. Cargo composition was further compared between vesicle populations, but only a paired whole-cell proteome from the same cultures will determine whether the observed differences reflect selective packaging rather than parent-cell composition. Lipidomic and RNA cargo profiling, particularly in Gram-negative species, should not be neglected, as they capture aspects of host interactions not accessible by proteomics alone and support a holistic interpretation of BMV influences. Nonetheless, this study provides a systematic cross-species comparison of BMVs from planktonic and biofilm cultures, showing that bacterial growth conditions profoundly shape vesicle composition and host cell interactions, highlighting key functional profiles, and further supporting the understanding of vesicular systems as a major force in bacterial life.

## Data Availability

The datasets presented in this study can be found in online repositories. The names of the repository/repositories and accession number(s) can be found in the article/**Supplementary Material**.

## Supplementary material

Supplementary Table 1 BMV yield, particle concentration, and particle-to-protein ratio. Protein concentrations were determined by bicinchoninic acid (BCA) assay on three independent isolations per sample group (N = 3, mean ± SD). For nanoparticle tracking analysis, the three isolations of each group were combined and diluted as indicated (also see 2.9). Particle concentrations of the preparation are the measured values multiplied by the dilution factor. Standard deviation for particle concentration and mean were reported by the instrument across the five captures; none of these values were compared statistically between sample groups.

Supplementary Table 2 BMVs were isolated from three independent planktonic and three independent biofilm cultures of each species (N = 3 per growth mode) and analyzed by LC-MS/MS with label-free quantification (MaxQuant v1.6.17.0, MaxLFQ). Spectra were searched against the species-specific UniProt reference proteomes (*P. aeruginosa* taxonomy ID 208964, 5,564 sequences; *S. aureus* taxonomy ID 93061, 2,889 sequences) together with a collection of common contaminants and filtered to a false discovery rate below 1% at peptide-spectrum-match and protein level. Protein groups without at least two of three valid values in one growth mode were removed; remaining missing values were imputed with default Perseus (v2.0.11) parameters, and log2 intensities were normalized by z-scoring. Growth modes were compared by Student’s t-test with Benjamini-Hochberg correction (BH). BH-adjusted p-value < 0.05 and difference in z-scored log2 intensity > 1 or < -1. Proteins meeting both criteria are marked “yes” in the “Differentially abundant” column and are highlighted in **Figure 4**. P*. aeruginosa*: 383 proteins reached adjusted p < 0.05 and 254 met the fold-change criterion; 230 met both (135 enriched in bBMVs, 95 in pBMVs). *S. aureus*: 509 reached adjusted p < 0.05 and 271 met the fold-change criterion; 259 met both (121 in bBMVs, 138 in pBMVs).
