# Supplementary material for: Proteomic profiling reveals growth condition-driven functional differences in bacterial membrane vesicles from planktonic and biofilm cultures
Source: Front Cell Infect Microbiol. 2026 Oct 1;16:1881620. doi: 10.3389/fcimb.2026.1881620 (PMC13668562; doi:10.3389/fcimb.2026.1881620)
Supplement: Supplementary file 1 [file Supplementaryfile1.docx]

Supplementary Material

# 1 Supplementary Figures and Tables

## 1.1 Supplementary Figures

**Figure S1. Biofilm characterization.** Histological analysis after hematoxylin staining of a fully colonized **(A)** *P. aeruginosa* biofilm (48 h) and **(B)** *S. aureus* biofilm (72 h). Representative section from N = 3 independently grown biofilms per organism. Scale bar represents 25 µm.

Figure S2. Stability testing of BMVs regarding hydrodynamic diameter and zeta potential. (A) Hydrodynamic diameter and (B) surface charge of *P. aeruginosa* and *S. aureus* BMVs immediately after isolation and after storage overnight at 4 °C. Bacteria were either grown as planktonic culture (blue) or biofilm (green) (N = 4). No statistical comparison was performed.

**Figure S3. Principal component analysis (PCA) of BMV proteome replicates.** PCA of log2-transformed intensities after filtering, imputation, and z-score normalization for BMVs from **(A)** *S. aureus* and **(B)** *P. aeruginosa*. Each circle represents one independent BMV isolation for either pBMVs (blue) or bBMVs (green) (N = 3 per growth mode). The first principal component separates the two growth modes in both species and accounts for 84.6 % (*S. aureus*) and 82.7 % (*P. aeruginosa*) of the total variance.

Figure S4. Baseline and endotoxin control responses of THP-1-derived macrophages. THP-1-derived M0 macrophages were incubated for 12 h and 24 h with medium alone (RPMI), polymyxin B alone (PMB, 30 µg/mL), LPS (100 ng/mL), or LPS + PMB. PMB was pre-incubated with LPS for 30 min at 37 °C prior to addition to the cells. (A) IL-1β, (B) IL-10, (C) IL-6, and (D) TNF-α in culture supernatants, quantified by ELISA and reported as absolute concentrations [pg/mL]. Data are plotted as mean ± SD (N = 3). Comparisons were carried out using a two-tailed Welch unequal-variance t-test with Bonferroni correction. * p < 0.05 after correction.

## 1.2 Supplementary Tables

**Supplementary Table S1.** BMV yield, particle concentration, and particle-to-protein ratio. Protein concentrations were determined by bicinchoninic acid (BCA) assay on three independent isolations per sample group (N = 3, mean ± SD). For nanoparticle tracking analysis, the three isolations of each group were combined and diluted as indicated (also see 2.9). Particle concentrations of the preparation are the measured values multiplied by the dilution factor. Standard deviation for particle concentration and mean were reported by the instrument across the five captures; none of these values were compared statistically between sample groups.

|  | ***S. aureus*** | | ***P. aeruginosa*** | |
| --- | --- | --- | --- | --- |
|  | **pBMVs** | **bBMVs** | **pBMVs** | **bBMVs** |
| **Protein concentration [µg/mL],  BCA (N = 3, mean ± SD)** | 999.62  ± 146.64 | 728.96  ± 380.34 | 685.84  ± 109.11 | 387.07  ± 32.01 |
| **Dilution factor for NTA** | 4 | 4 | 2 | 2 |
| **Particle concentration in the measured dilution [mL⁻¹]  (combined preparation)** | 1.11 × 10⁹  ± 4.06 × 10⁸ | 9.96 × 10⁸  ± 8.08 × 10⁷ | 2.07 × 10⁹  ± 6.05 × 10⁸ | 1.03 × 10⁹  ± 2.88 × 10⁸ |
| **Particle concentration of the preparation [mL⁻¹]  (combined preparation)** | 4.44 × 10⁹ | 3.98 × 10⁹ | 4.14 × 10⁹ | 2.06 × 10⁹ |
| **Particles per µg protein  (combined preparation)** | 4.44 × 10⁶ | 5.46 × 10⁶ | 6.04 × 10⁶ | 5.32 × 10⁶ |
| **Modal diameter [nm]** | 117.5 | 122.5 | 132.5 | 112.5 |
| **Mean diameter [nm]  (± SD of n = 5)** | 180 ± 23 | 211 ± 9 | 164 ± 18 | 148 ± 14 |
| **Valid tracks** | 23,400 | 16,197 | 27,408 | 21,466 |

**Supplementary Table S2.** BMVs were isolated from three independent planktonic and three independent biofilm cultures of each species (N = 3 per growth mode) and analyzed by LC-MS/MS with label-free quantification (MaxQuant v1.6.17.0, MaxLFQ). Spectra were searched against the species-specific UniProt reference proteomes (*P. aeruginosa* taxonomy ID 208964, 5,564 sequences; *S. aureus* taxonomy ID 93061, 2,889 sequences) together with a collection of common contaminants and filtered to a false discovery rate below 1 % at peptide-spectrum-match and protein level. Protein groups without at least two of three valid values in one growth mode were removed; remaining missing values were imputed with default Perseus (v2.0.11) parameters, and log2 intensities were normalized by z-scoring. Growth modes were compared by Student's t-test with Benjamini-Hochberg correction (BH). BH-adjusted p-value < 0.05 and difference in z-scored log2 intensity > 1 or < -1. Proteins meeting both criteria are marked "yes" in the “Differentially abundant” column and are highlighted in Figure 4. *P. aeruginosa*: 383 proteins reached adjusted p < 0.05 and 254 met the fold-change criterion; 230 met both (135 enriched in bBMVs, 95 in pBMVs). *S. aureus*: 509 reached adjusted p < 0.05 and 271 met the fold-change criterion; 259 met both (121 in bBMVs, 138 in pBMVs).
